# Core and Shell Song Systems Unique to the Parrot Brain

**DOI:** 10.1371/journal.pone.0118496

**Published:** 2015-06-24

**Authors:** Mukta Chakraborty, Solveig Walløe, Signe Nedergaard, Emma E. Fridel, Torben Dabelsteen, Bente Pakkenberg, Mads F. Bertelsen, Gerry M. Dorrestein, Steven E. Brauth, Sarah E. Durand, Erich D. Jarvis

**Affiliations:** 1 Department of Neurobiology, Duke University Medical Center, Durham, NC, United States of America; 2 Howard Hughes Medical Institute, Chevy Chase, Maryland, United States of America; 3 Department of Biology, University of Copenhagen, Copenhagen, Denmark; 4 Danish National Police, National Centre of Forensic Services, Vanloese, Denmark; 5 Research Laboratory for Stereology and Neuroscience, Bispebjerg University Hospital, Copenhagen, Denmark; 6 Copenhagen Zoo, Frederiksberg, Denmark; 7 Dutch Research Institute of Avian and Exotic Animals, Veldhoven, The Netherlands; 8 University of Maryland, College Park, MA, United States of America; 9 LaGuardia Community College, New York, NY, United States of America; UCLA, UNITED STATES

## Abstract

The ability to imitate complex sounds is rare, and among birds has been found only in parrots, songbirds, and hummingbirds. Parrots exhibit the most advanced vocal mimicry among non-human animals. A few studies have noted differences in connectivity, brain position and shape in the vocal learning systems of parrots relative to songbirds and hummingbirds. However, only one parrot species, the budgerigar, has been examined and no differences in the presence of song system structures were found with other avian vocal learners. Motivated by questions of whether there are important differences in the vocal systems of parrots relative to other vocal learners, we used specialized constitutive gene expression, singing-driven gene expression, and neural connectivity tracing experiments to further characterize the song system of budgerigars and/or other parrots. We found that the parrot brain uniquely contains a song system within a song system. The parrot “core” song system is similar to the song systems of songbirds and hummingbirds, whereas the “shell” song system is unique to parrots. The core with only rudimentary shell regions were found in the New Zealand kea, representing one of the only living species at a basal divergence with all other parrots, implying that parrots evolved vocal learning systems at least 29 million years ago. Relative size differences in the core and shell regions occur among species, which we suggest could be related to species differences in vocal and cognitive abilities.

## Introduction

Vocal learning, a critical behavior for spoken language, is the ability to modify acoustic and/or syntactic features of sounds produced, including improvisation and imitation. Complex vocal learning has so far been found in five distantly related groups of mammals (humans, bats, elephants, cetaceans [dolphins and whales], and pinnipeds [seals and sea lions]) and three distantly related groups of birds (parrots, songbirds, and hummingbirds) [1–5]. Vocal learning is thought to have evolved to allow for more complex communication and cultural transmission of learned conspecific vocal repertoires that are important for social cohesion [2,6]. Because complex vocal learning is not found in their close relatives, it is generally thought that each vocal learning lineage evolved this trait independently [1,2]. Recent studies have placed parrots as a sister group to passeriform songbirds [7,8], leading to an alternative interpretation of a common origin of complex vocal learning in parrots and oscine songbirds, followed by two subsequent losses in suboscines and New Zealand Wrens.

Interestingly, not all vocal learners display vocal mimicry, defined as the ability to imitate heterospecific vocalizations [9–13]. Species that readily imitate other species include parrots, such as the African Grey (*Psittacus erithacus*), and corvid songbirds, such as crows and the Greater Indian Hill Mynahs (*Gracula religiosa intermedia*), and humans [14–16]. Some vocal learning bird species have a remarkable ability to imitate rudimentary human speech [10,15,17,18]. Vocal mimicry has also been observed in some mammalian vocal learners, including speech or whistle imitation in bottlenose dolphins (*Tursiops truncatus*), killer whales (*Orcinus orca*), harbor seals (*Phoca vitulina*), and elephants [13,19–23], but none have been reported to have the ability to mimic human speech sounds as well as parrots.

**Fig 1:**
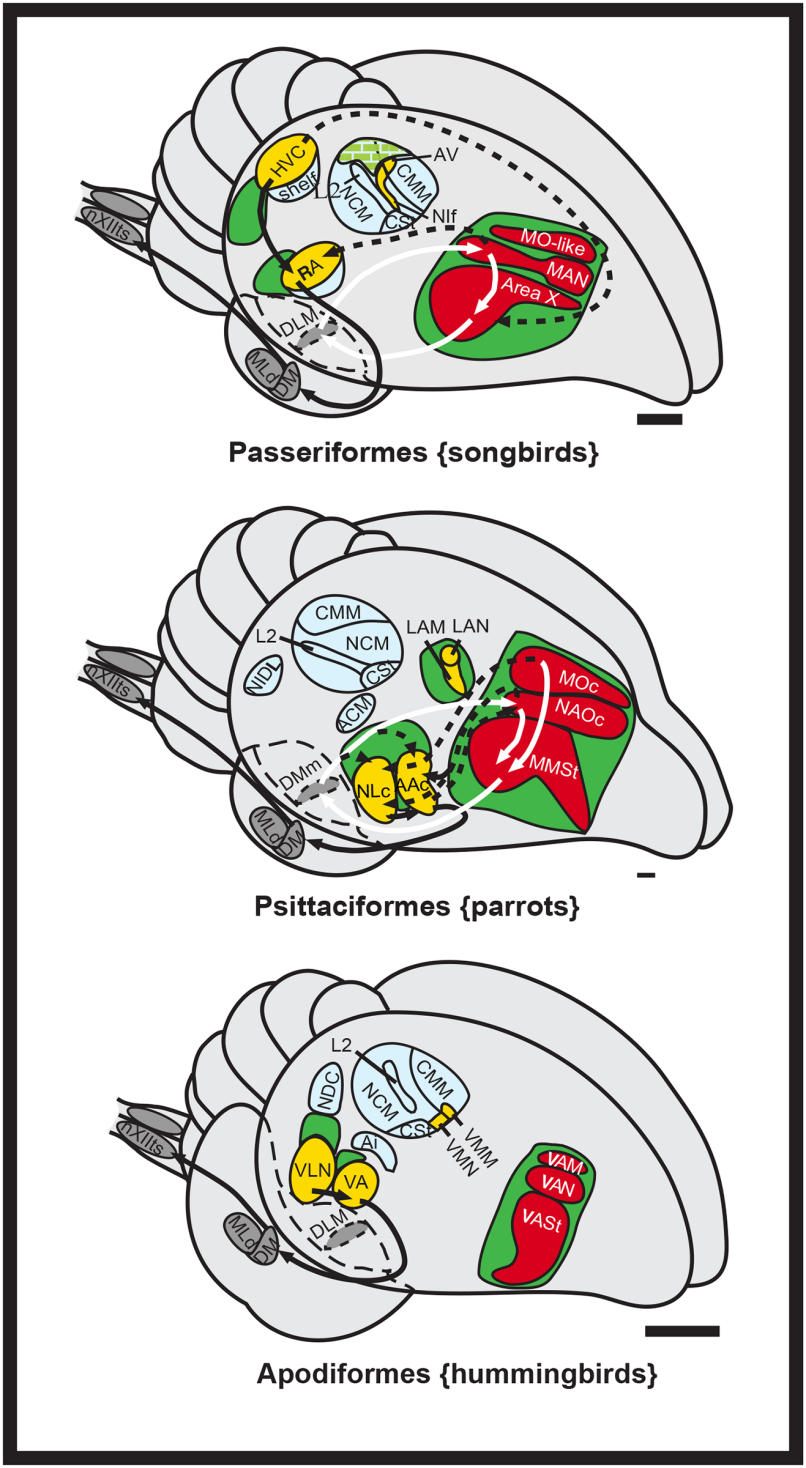
Song systems of avian vocal learners. Shown is a schematic 3D drawing of seven cerebral vocal nuclei (red and yellow) found in vocal learners, and auditory areas (blue) and movement associated areas (green) found in all birds. Checkered green is both movement and hearing activated regions; red vocal nuclei and white arrows, anterior vocal pathway; yellow-labeled vocal nuclei and black arrows, posterior vocal pathway. Only a few connections in hummingbirds are known and that of songbird MO is not known. Figure modified from Feenders et al. [35] and Jarvis et al. [27]. Scale bars, 1 mm. See list for Abbreviations.

The neural pathways for vocal learning have been characterized in multiple songbird and hummingbird species, but in only one parrot species, the budgerigar (*Melopsittacus undulatus*) [1,24–29]. In these species, immediate early genes (IEGs), tracers, brain region inactivation and electrophysiology recordings (with the exception in hummingbirds) have been used to identify up to seven cerebral (telencephalic) vocal nuclei each, which are considered analogous in all three avian lineages (Fig 1, yellow and red [24–27,30–34]). The seven cerebral song nuclei are distributed within two pathways [24,27,30] best characterized in songbirds as: (1) an anterior song pathway that forms a pallial-basal-ganglia-thalamic loop and influences vocal learning, syntax, and social context functions (Fig 1, red); and (2) a posterior song pathway that influences the production of learned song (Fig 1, yellow). In all three groups, the song nuclei of both pathways are embedded within or adjacent to non-vocal movement activated brain regions (Fig 1, green), a finding that led to the motor theory of vocal learning origin, where vocal learning pathways are proposed to have evolved from a pre-existing ancestral motor learning pathway shared by vertebrates [35].

Thus far, differences found between vocal learning bird species include that the posterior vocal motor pathway is also anatomically adjacent to the auditory pathway in songbirds, but more distant to it in hummingbirds and parrots (Fig 1, blue) [24,27]. The connectivity between the posterior and anterior pathways is more different between songbirds and parrots than within each subpathway (tracing experiments in hummingbirds are not extensive) [5]. Another difference is that besides the caudal auditory pathway regions’ (e.g. Field L) input into the posterior vocal pathway as in songbirds, in the parrot, auditory neurons within the somatosensory nucleus basorostralis [B] also send input into the overlying posterior vocal pathway song nucleus LAN (lateral nucleus of the anterior nidopallium; analog of songbird NIf, interfacial nucleus of the nidopallium) [24,32,36]. However, the tracing studies in budgerigars have only identified a sub-region of the anterior vocal nuclei activated by vocalizing-driven gene expression, called the core anterior nuclei in Jarvis and Mello [24]. The vocalizing-driven gene expression studies revealed a surround region of activation for the anterior pathway nuclei, not specifically noted in tracing studies published before or after. Further, the parrot posterior vocal motor pathway nuclei NLC (central nucleus of the lateral nidopallium) and AAC (central nucleus of the anterior arcopallium) contain anatomically distinct dorsal and ventral subdivisions as described in tracing studies [32,34] that also show vocalizing-induced gene expression [24,35]. However, the areas surrounding the NLC described as the “shell”, “NLs” (supracentral nucleus of the lateral nidopallium), and “NLv” (ventral lateral nidopallium) in Durand et al. [34] appear to encompass the motor areas surrounding the NLC described in Feenders et al. [35]. This has caused considerable confusion in the field as to the organization and terminology of the parrot song nuclei and attempts to resolve the differences and similarities have been few [24,31,34]. Although, differences in connectivity, position, or shape of song nuclei have been noted between parrots compared to songbirds and hummingbirds [5,24,32,34], no differences have been noted in the presence or absence of song system nuclei.

To better understand the organization of the song system in parrots, its evolution, and specialized molecular properties relative to other vocal learning avian species, we performed analyses of brain gene expression profiles in nine different parrot species representing all the three superfamilies, Strigopoidea, Cacatuoidea, and Psittacoidea, for genes with known specialized expression in vocal learning nuclei, and compared those findings with vocalizing-driven gene expression and neural tracing experiments. The set of genes that show specialized expression, meaning higher or lower expression in the song nuclei compared to the surrounding regions, include among many others Parvalbumin (*PVALB*), glutamate receptors, *FOXP1*, and *SLIT1* [37–39]. These analyses led us to hypothesize that the parrot vocal learning system contains “core” and “shell” region specializations, with the core system similar to the song system of songbirds and hummingbirds and the shell system unique to parrots. This dual system evolved early in the parrot lineage and has lasted and expanded for millions of years in different species.

## Results

We performed a series of experiments that led us to conclude that brain regions that include song nuclei of parrots have three concentric levels of specializations: (1) the inner core song nuclei; (2) the outer shell song nuclei that surround the cores; and (3) the outer non-vocal motor regions that surround the song nuclei shells. We described our more in depth findings in budgerigars first, and then comparisons with other parrot species.

### Gene expression profiles reveal core and shell regions within the cortical-like song nuclei of budgerigars

**Fig 2:**
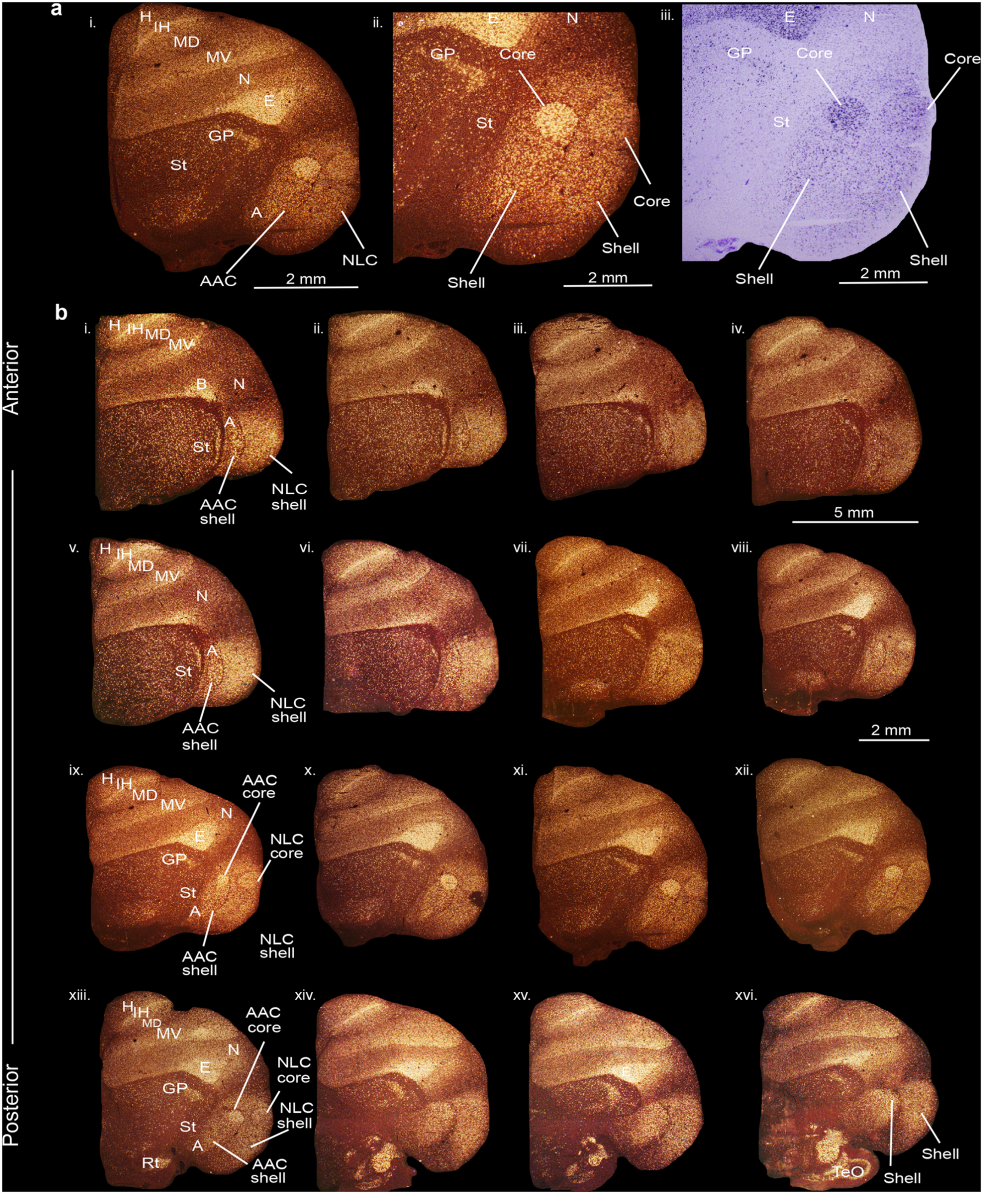
*PVALB* mRNA expression in the core and shell regions of the NLC and AAC song nuclei in the budgerigar brain. (**a**) High power view of darkfield (i-ii) and brightfield (cresyl violet stained) (iii) images showing expression of parvalbumin mRNA. (**b**) Serial sections of a budgerigar brain (i-xvi) showing the core and shell regions of NLC and AAC song nuclei. Silver grains is white; cresyl violet is red-brown. Sections are in the coronal plane; medial is to the left, dorsal is top. See list for abbreviations.

We first performed anatomical gene expression analyses for the *PVALB* gene, which generates a calcium-binding protein with a role in neuroprotection and critical period plasticity, preferentially in GABA-expressing interneurons [40,41]. In contrast to our prior study [38], here we processed sections for *PVALB* mRNA expression serially throughout the budgerigar forebrain. We found that *PVALB* expression revealed core (very high) and shell (moderate) regions of expression that included the posterior song nuclei NLC and AAC and surrounding motor areas relative to the surrounding nidopallium (N) and arcopallium (A), respectively (Fig 2a; serial sections in 2b). The *PVALB-*defined cores were shaped like spheres, had Nissl-defined boundaries, were positioned dorsally within the shells, and were largest in size in sections in the middle of the song nuclei (Fig 2b, sections viii to xiii; S1 Fig), gradually diminishing in size in the anterior and posterior directions. The *PVALB-defined* shells surrounded the cores dorsally and ventrally (Fig 2a). The shell regions were much larger in ventral, anterior, and posterior directions, than dorsally (Fig 2b, sections i to vii and xiv to xvi). The boundaries of the *PVALB*-defined shells were not detectable in Nissl staining, but within them we noted less prominent Nissl-defined boundaries, which correspond to what we call in this study shell song nuclei (Fig 2; S1 Fig).

We also noted higher *PVALB* expression in the primary sensory neural populations of the telencephalon and thalamus. These included the somatosensory basorostralis [B], visual entopallium [E], auditory L2 [not shown], and visual intercalated hyperpallium [IH] in the telencephalon and the visual nucleus rotundus (Rt) in the thalamus. There was also higher expression at the boundaries of the ventral mesopallium (MV) with the adjacent nidopallium and the dorsal mesopallium (MD) with the intercalated hyperpallium (Fig 2a and 2b; terminology is according to a recently revised nomenclature and understanding of avian brain organization [42,43]).

**Fig 3:**
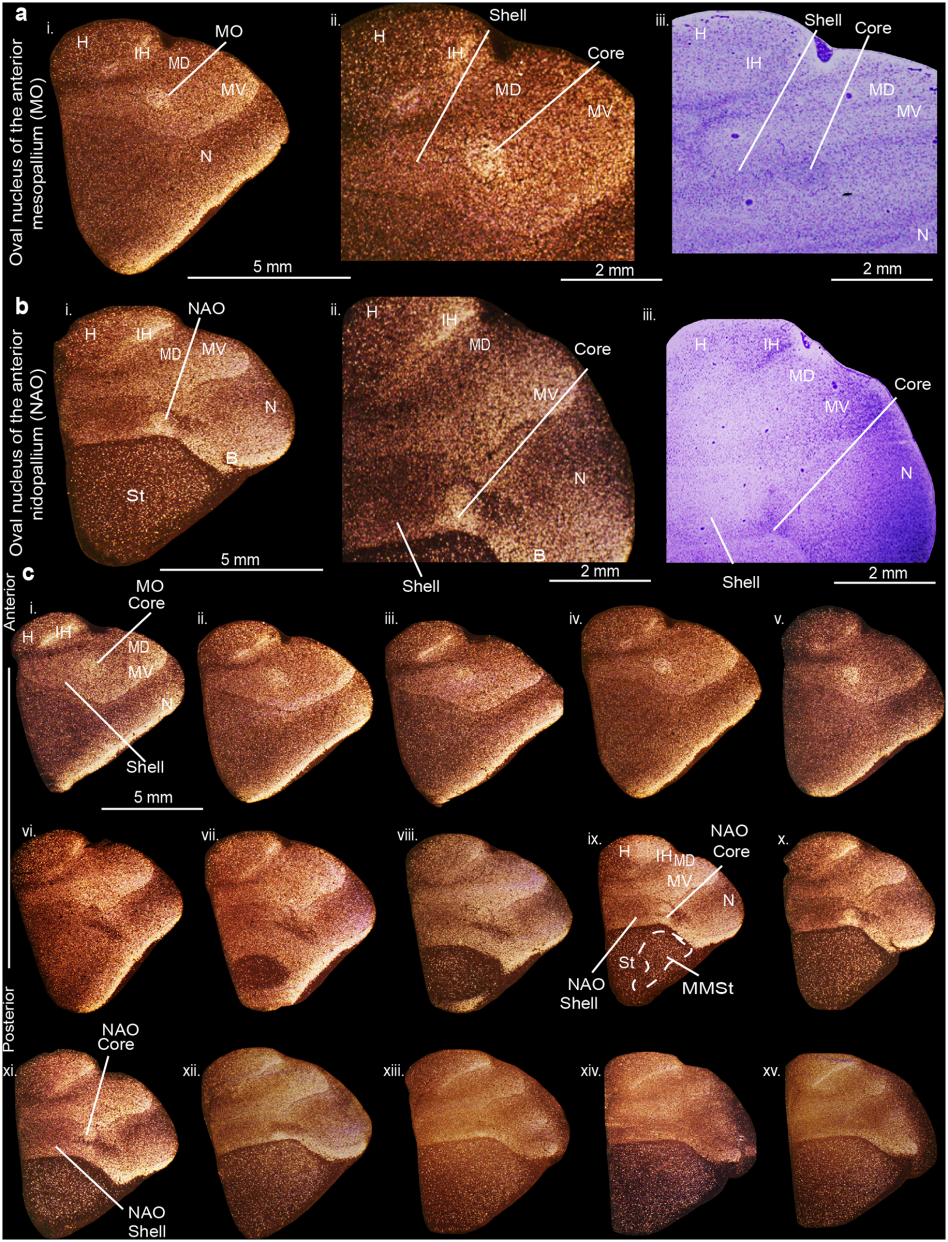
*PVALB* mRNA expression in the core and shell regions of the MO and NAO song nuclei in the budgerigar brain. (**a**) High power view of darkfield (i-ii) and brightfield (cresyl violet stained) (iii) images showing expression of parvalbumin mRNA in MO. (**b**) High power view of darkfield (i-ii) and brightfield (cresyl violet stained) (iii) images showing expression of parvalbumin mRNA in NAO. (**c**) Serial sections of a budgerigar brain (i-xv) showing the core and shell regions of MO and NAO song nuclei. Silver grains is white; cresyl violet is red-brown. Sections are in the coronal plane; medial is to the left, dorsal is top. See list for abbreviations.

In contrast to the posterior song nuclei, we discovered high *PVALB* expression in only the well-defined core regions of the anterior song nuclei MO (oval nucleus of the mesopallium) and NAO (oval nucleus of the anterior nidopallium) (Fig 3a–3c). The MO core nucleus was distinguishable with Nissl staining, shaped like an egg sitting on its side, with its narrower end pointed towards the midline, comprised of large, loosely distributed cells relative to the surrounding mesopallium (S2 Fig). The NAO core was positioned more anterior and sat directly adjacent to the somatosensory basorostralis also of higher *PVALB* expression. The MO and NAO shells, like NLC and AAC shells, were partly distinguishable in Nissl from the surrounding motor mesopallium and nidopallium, respectively, by their shared cellular orientation (S2 Fig) as previously noted [31,32,34]. We did not detect core and shell region specializations of *PVALB* expression within the striatal song nucleus, MMSt (magnocellular nucleus of the medial striatum). However, we noted a uniformly moderate higher expression than the surrounding striatum (St) in sections that spanned the MMSt song nucleus rostro-caudally (Fig 3c, sections ix to xv).

### Functional activation of core and shell regions

To determine the functional makeup of the *PVALB*-defined core and shell regions, and whether core and shell song nuclei regions were confined to the Nissl-defined boundaries we identified, we performed side-by-side comparisons of comparable serial brain sections that we had collected with vocalizing-driven (warble song) and non-singing motor-driven (hopping) *DUSP1*, *EGR-1* (also known as ZENK), and *C-FOS* IEG expression [24,35,44,45]. *DUSP1* is an activity-dependent dual sensitivity phosphatase that shows motor-driven gene expression only in song nuclei of vocal learners and sensory-driven expression in the primary sensory neurons in all species [45], whereas *EGR-1* and *C-FOS* are activity-dependent transcription factors that show behaviorally-driven expression in many cell types, including in song nuclei and the surrounding motor and auditory regions [24,35,44,46].

**Fig 4:**
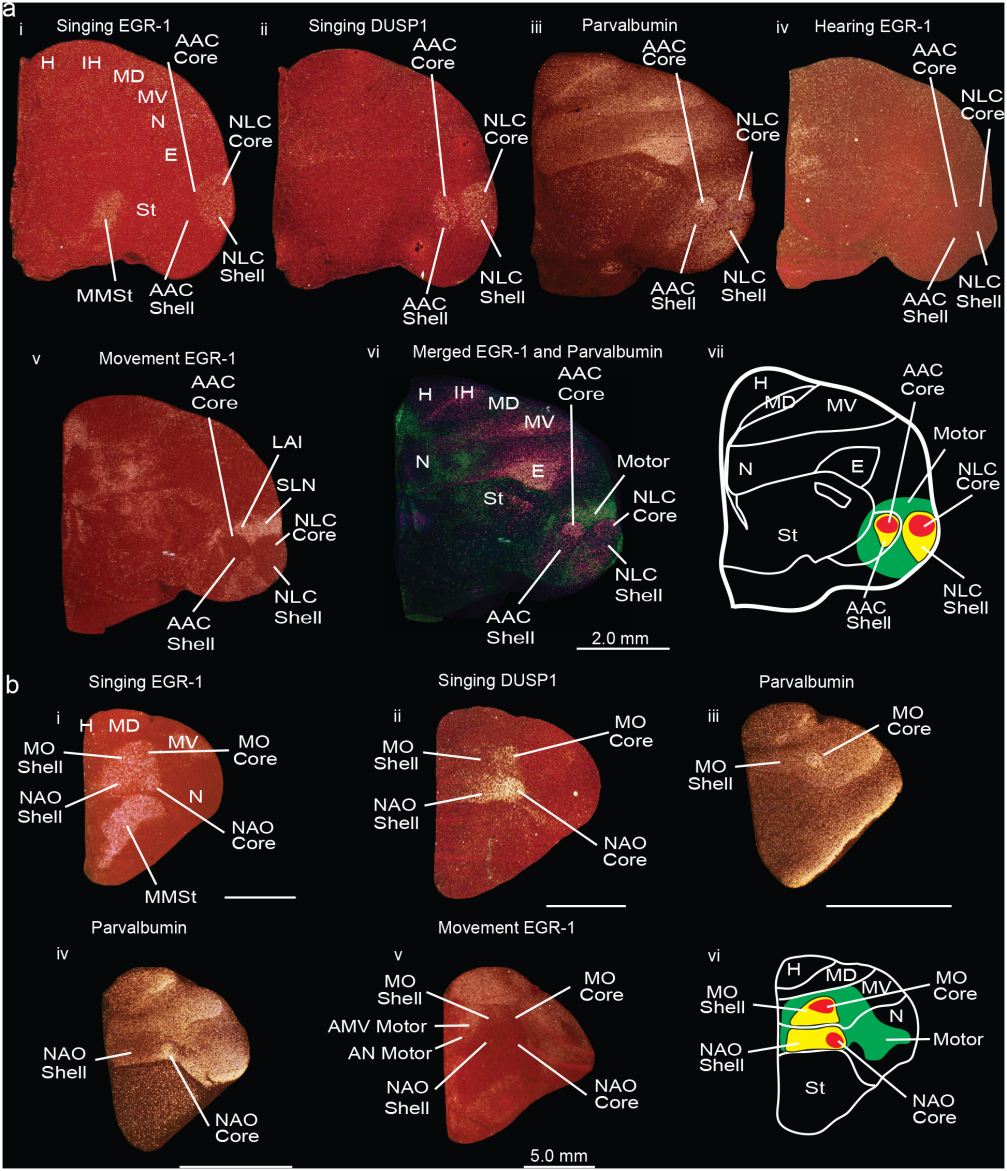
Darkfield photomicrographs showing *PVALB*, *EGR-1*, and *DUSP1* mRNA expression in the budgerigar brain in different behavioral states. (**a**) Darkfield images of *EGR-1*, *DUSP1*, and *PVALB* mRNA expression in sections containing song nuclei of the posterior vocal pathway. (i) Vocalizing-induced *EGR-1* expression within the NLC core and shell; *EGR-1* mRNA expression is not distinguishable in AAC in this animal because of lower amount of vocalizing behavior; (ii) Vocalizing-induced *DUSP1* expression in the NLC and AAC core and shells; (iii) *PVALB* specialized expression in the NLC and AAC core and shells, as well as adjacent motor areas around the shells; (iv) Lack of hearing-induced *EGR-1* expression in the NLC and AAC in a silent bird; (v) Movement-induced *EGR-1* expression in regions around the NLC shell and AAC shell song nuclei; (vi) Merged images of movement-induced *EGR-1* expression (green) which overlaps with the motor part of the shell region defined by *PVALB* expression (purple), and the *PVALB*-defined core and shell regions of NLC and AAC (purple); (vii) Schematic diagram showing regions of song nuclei cores (red) and shells (yellow), and adjacent motor shell regions (green). (**b**) Darkfield photomicrographs of *PVALB*, *EGR-1*, and *DUSP1* mRNA expression in coronal brain sections containing anterior vocal pathway song nuclei. (i) Vocalizing-induced *EGR-1* expression in the MO and NAO core and shell song nuclei, and the MMSt song nucleus; (ii) Vocalizing-induced *DUSP1* expression in the MO and NAO core and part of shell song nuclei; (iii) *PVALB* expression in the MO core; (iv); *PVALB* expression in the NAO core; (v) Movement-induced *EGR-1* expression in the areas surrounding the MO and NAO shells; (vi) Schematic diagram showing regions of the MO and NAO core (in red) and shell (yellow) song nuclei, and motor regions surrounding them (green). Sections are in the coronal plane; medial is to the left, dorsal is top. Some sections are compiled from the same animals of studies conducted in Horita et al. [45] (panels a-i to ii, b-ii), Jarvis and Mello [24] (a-iv, b-i), and Feenders et al. [35] (panels a-v, b-v). See Table 3 for nomenclature. See list for Abbreviations.

**Fig 5:**
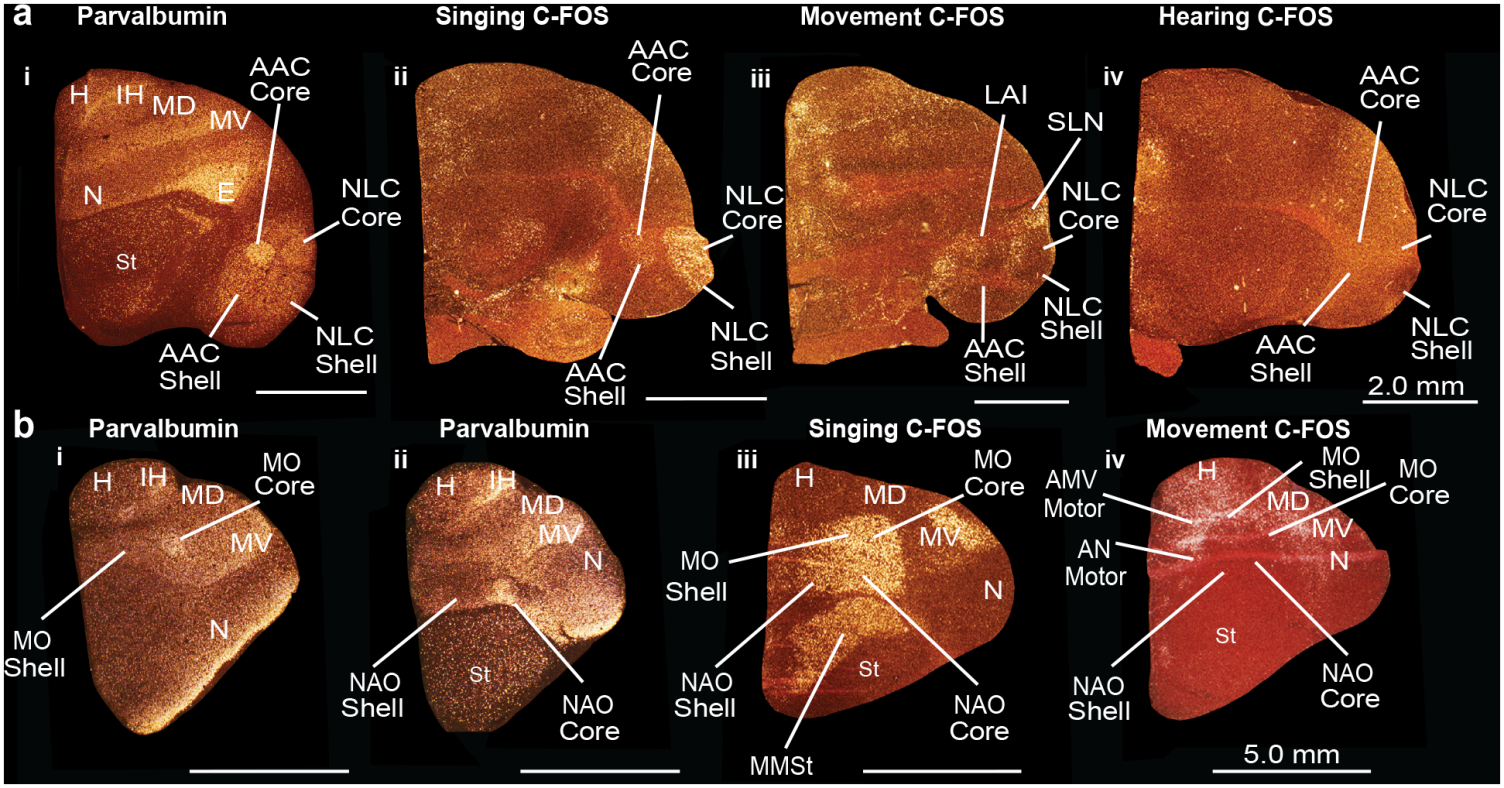
Darkfield photomicrographs showing *PVALB* and *C-FOS* mRNA expression in the budgerigar brain in different behavioral states. (**a**) *PVALB* and *C-FOS* mRNA expression in the NLC and AAC core and shells. (i) *PVALB* specialized mRNA expression in the NLC and AAC core and shell, as well as adjacent motor areas; (ii) Vocalizing-driven *C-FOS* mRNA expression in the NLC and AAC core and shells; (iii) Movement-induced *C-FOS* mRNA expression in parts of the shell regions around NLC and AAC; (iv) Lack of hearing-induced *C-FOS* mRNA expression in the NLC and AAC song nuclei in a silent bird. (**b**) *PVALB* and *C-FOS* mRNA expression in the MO and NAO of the anterior vocal pathway in birds. (i) *PVALB* mRNA expression in the MO core; (ii) *PVALB* mRNA expression in the NAO core; (iii) Vocalizing-induced *C-FOS* mRNA expression in the MO and NAO core and shells, and the MMSt song nucleus; (iv) Movement-induced *C-FOS* mRNA expression in the areas surrounding the MO and NAO core and shells, and the MMSt song nucleus. Sections are in the coronal plane; medial is to the left, dorsal is top. Some sections are from the same animals used in experiments of Feenders et al. [35] (a-iii, b-iv), Jarvis and Mello [24], and Jarvis [44] (a-ii, a-iv, b-iii, b-iv). See list for Abbreviations.

We found that the functionally defined, vocalizing-driven *EGR-1* and *C-FOS* boundaries encompassed the entire *PVALB*-defined cores and part of the *PVALB*-defined shells of the NLC and AAC (Fig 4a, sections i, iii; Fig 5a, sections i-ii). The vocalizing-driven *DUSP1* expression overlapped similarly with *PVALB*, with higher expression in AAC than *EGR-1* or *C-FOS* (Fig 4a, sections ii-ii). The remainder of the *PVALB*-defined shells extended beyond the vocalizing-driven functionally-defined boundaries of the NLC and AAC, and overlapped with the non-vocal movement-driven *EGR-1* and *C-FOS* gene expression regions, including what has been called the supra lateral nidopallium (SLN) around the NLC shell and the lateral intermediate arcopallium (LAI) around the AAC shell (Fig 4a, sections v-vi; Fig 5a, sections i-iii). In animals that heard warble song but did not vocalize and sat relatively still, there was no detectable *EGR-1* or *C-FOS (DUSP1 not shown)* induction in the *PVALB*-defined song nuclei and surround (Fig 4a, section iv; Fig 5a, section iv). Therefore, we conclude that there are two levels of *PVALB*-defined specializations involving the posterior NLC and AAC song nuclei: the *PVALB*-defined song nuclei cores (high expression) and shells (moderate to low expression) that are vocally active; and the remainder of the *PVALB*-defined shells (moderate expression) that are non-vocal motor active regions (Fig 4a, section vii).

In the anterior song nuclei MO and NAO, the functionally defined, vocalizing-driven *EGR-1* and *C-FOS* regions encompassed the entire *PVALB*-defined core (Fig 4b, sections i,iii,iv; Fig 5b, sections i-iii). The vocalizing-driven *EGR-1* and *C-FOS* expression defined the boundaries of the song nuclei shells (Fig 4b, section i) with the movement-driven expression-defined regions of the anterior ventral mesopallium (AMV) and anterior nidopallium (AN) (Fig 4b, section v). Vocalizing-driven *DUSP1* expression was highest in the cores but exhibited lower expression in the anterior song nuclei shells (Fig 4b, section ii). Therefore, in contrast to the posterior song nuclei, there is only one level of *PVALB*-defined specialization involving the anterior song nuclei: the *PVALB*-defined cores (high expression). The anterior song nuclei shell regions share non-specialized *PVALB* expression with the surrounding motor regions (Fig 4b, section vi).

### Songbirds and hummingbirds do not have shell song nuclei

Core versus shell gene expression specializations have not been described in the ‘vocal-motor active’ brain regions of songbirds or hummingbirds. To validate whether the vocal-motor pattern we found is unique to parrots, we examined published and previously unpublished serial sections containing song nuclei that were hybridized for *PVALB* and for vocalizing-driven *DUSP1*, *C-FOS*, or *EGR-1* expression from our studies in songbirds and hummingbirds [35,38,44,45] namely the zebra finch (*Taeniopygia guttata*) and the Anna’s hummingbird (*Calypte anna)*, respectively.

**Fig 6:**
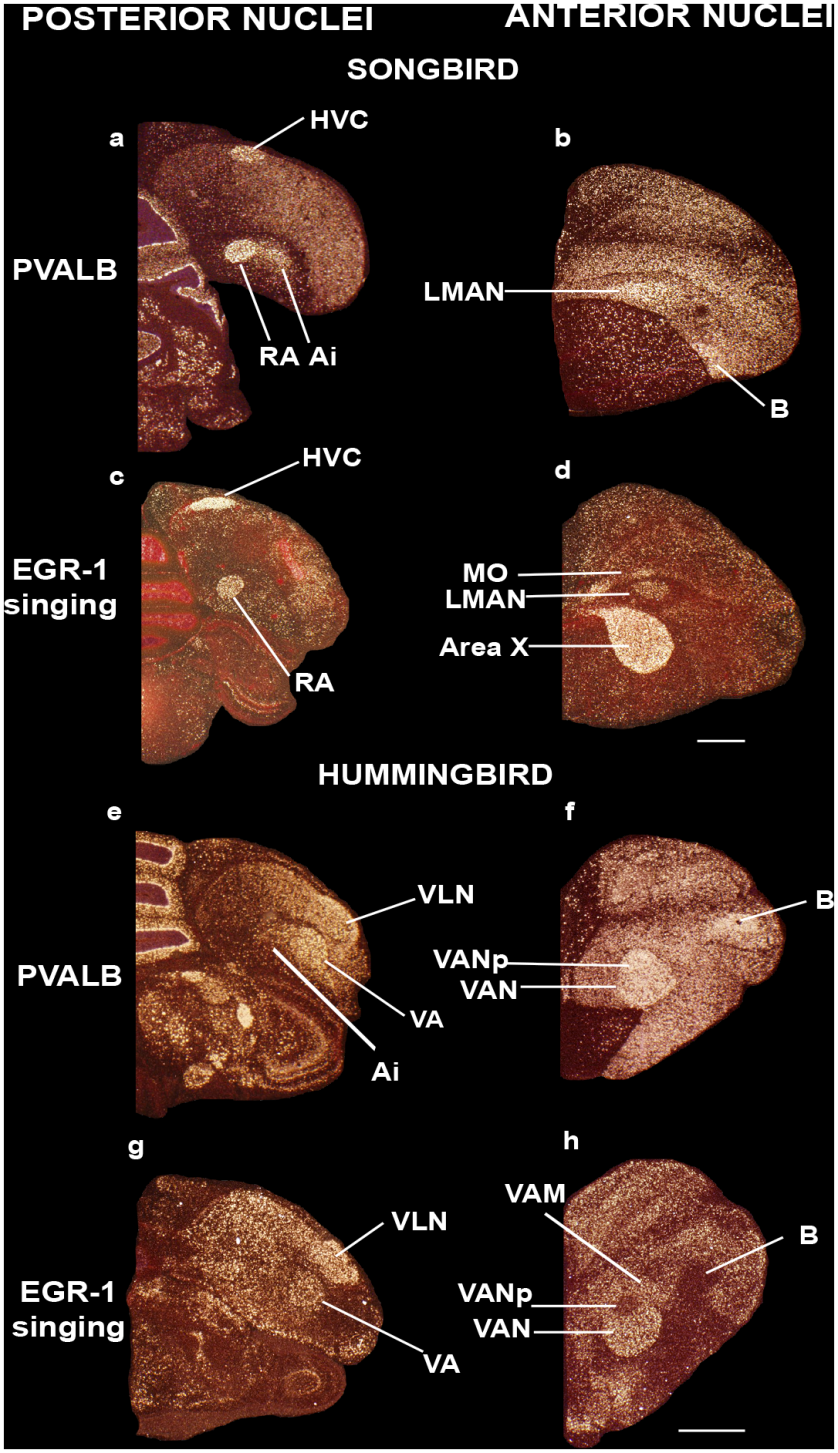
Darkfield photomicrographs showing *PVALB* and *ZENK* mRNA expression in male zebra finch (a songbird) and Anna’s hummingbird brains. (**a**) High *PVALB* expression in HVC and RA song nuclei, and moderate expression in Ai in the male zebra finch; (**b**) *PVALB* expression in LMAN song nucleus in the zebra finch; (**c**) Vocalizing-driven *EGR1* expression in HVC and RA song nuclei in a male zebra finch that was singing alone in a sound box; (**d**) Vocalizing-driven *EGR1* expression in MO, LMAN, and Area X song nuclei of the same zebra finch as in (c); (**e**) *PVALB* expression in VLN and VA song nuclei of the male Anna’s hummingbird; (**f**) High *PVALB* expression in VAN in the Anna’s hummingbird not vocalizing; (**g**) Vocalizing-driven *EGR1* expression in VLN and VA song nuclei of the Anna’s hummingbird that was also flying and feeding, in the early morning (1h after sunrise); (**h**) Vocalizing-driven *EGR1* expression in the VAN song nucleus, except within an internal region dorsal posterior region (VANp). Sections are in the coronal plane; medial is to the left, dorsal is top. Panels are sections collected from animals used in Hara et al. [38] (panels a, b, e, f) and Feenders et al. [35] (c, d, g, h). See list for Abbreviations. Scale bars = 2mm.

In the zebra finch we did not find evidence of differential core and shell *PVALB* expression “within” the song nuclei. Instead, in support of previous conclusions [38], the differential *PVALB* expression was uniformly high throughout each song nucleus (Fig 6a and 6b), and restricted to the vocalizing-driven gene expression defined boundaries (Fig 6c and 6d); these were HVC (a vocal nucleus, no abbreviation), RA (robust nucleus of the arcopallium), and LMAN (lateral part of the magnocellular nucleus of the anterior nidopallium), analogs of parrot NLC, AAC, and NAO, respectively. We did not note differential *PVALB* expression within the MO analog of songbirds in the mesopallium, which is relatively much smaller in songbirds (Fig 6b and 6d). Previous studies have characterized areas surrounding songbird song nuclei such as the HVC shelf, LMAN shell, and RA cup, parts of which are known to be auditory [47–50] and/or non-vocal motor [35,45] in function, or proposed to be accessory to the song system [51,52]. In this regard, we noted that the banana-shaped intermediate lateral arcopallium (LAI) directly adjacent to RA, part of which controls movement [35,50], had moderately higher levels of *PVALB* expression relative to the remaining arcopallium (Fig 6a) similar to the non-vocal motor LAI regions adjacent to the parrot AAC shell. We did not note any additional *PVALB* specializations restricted to the non-vocal HVC shelf or LMAN shell.

In the hummingbird, as in songbirds, differential *PVALB* expression in each song nucleus was uniformly high relative to the immediate adjacent surrounding non-song regions (Fig 6e and 6f); these were VLN (vocal nucleus of the lateral nidopallium), VA (vocal nucleus of the arcopallium), and VAN (vocal nucleus of the anterior nidopallium), analogs of parrot NLC, AAC, and NAO, respectively. *PVALB* expression was restricted to the vocalizing-driven gene expression defined boundaries (Fig 6g and 6h), and not present in VAM (vocal nucleus of the anterior mesopallium), analog of parrot MO (Fig 6f and 6h). As in songbirds and parrots, the immediate surrounding Ai (intermediate arcopallium) region that includes the movement activated part (LAI) [35] exhibited intermediate *PVALB* levels (Figs 4, 6e). In contrast, the hummingbird VAN (budgerigar NAO analog) includes a dorsocaudal region lacking vocalizing-driven *EGR-1* expression (Fig 6h) as noted previously [27]. But this region exhibits higher *PVALB* expression (Fig 6f), similar to the more anterior and larger sized vocalization-driven *EGR-1* expression part (Fig 6h). Overall, these findings suggest that the parrot shell song regions are unique among avian vocal learners.

### Multiple genes show shell and core region specializations in budgerigar

We wondered if our findings were specific to *PVALB* or would other genes that are differentially expressed in song nuclei of songbirds also show differential expression in core versus shell regions of parrots. Including *PVALB*, we identified 10 genes from the past and recent literature, including that of our own studies, with differential mRNA or protein expression in core versus shell song nuclei or adjacent non-vocal motor regions of budgerigars (Table 1).

**Table 1:** Summary of gene expression specializations in budgerigar.

	NLC core	NLC shell	NLC motor	AAC core	AAC shell	AAC motor	MO core	MO shell	NAO core	NAO shell	MMSt
**Baseline**											
PVALB	+++	++	++	+++	++	++	+++		+++		+
SLIT1				---							
FOXP1	++	+									
NR2A	+++	+									
GLUR1	--										
NADPH-d	+++	++	++	+++	++	++	+++		+++	+	++
AR	+++	+++		+++	+++		++		+++	++	
mENK fiber	+++	++		+++	++	++	+++		+++	+	++
mENK cell	++	++		++							
TH fiber	--	-		--	--		--	-	-		+
CGRP-LI fibers	++	++		+	+		+++		+++	+	+
**Activity**											
DUSP1	+++	+++		+++	+++		+++	+	+++	++	
C-FOS	+++	+++	+++	++	++	++	+++	+++	+++	+++	+++
EGR1	+++	+++	+++	+	+	+	+++	+++	+++	+++	+++

The data represent qualitative analyses of gene expression (mRNA or protein) labeling in the song nucleus relative to the surround of that same brain subdivision conducted in this and other studies. + = Relative level of expression higher than the surrounding non-vocal or non-motor areas;— = lower expression; no value entered means no difference relative to the surrounding areas. The other studies are: *PVALB*, Hara et al. [38]; *SLIT1*, Wang et al. [39]; *FOXP1*, Haesler et al. [54]; *EGR-1*, Feenders et al. [35]; *NR2A*, Wada et al. [56]; *GLUR1*, Wada et al. [56]; NADPH-d, Brauth et al. [57]; *AR*, Matsunaga and Okanoya [60]; mENK, Durand et al. [58]; TH, Roberts et al. [61]; CGRP-LI, Durand et al. [59]; *DUSP1*, Horita et al. [45], *C-FOS*, Jarvis [44]; *EGR-1*, Jarvis and Mello [24]. The *NR2A* mRNA levels are also consistent with protein levels detected by immunocytochemistry [85].

**Fig 7:**
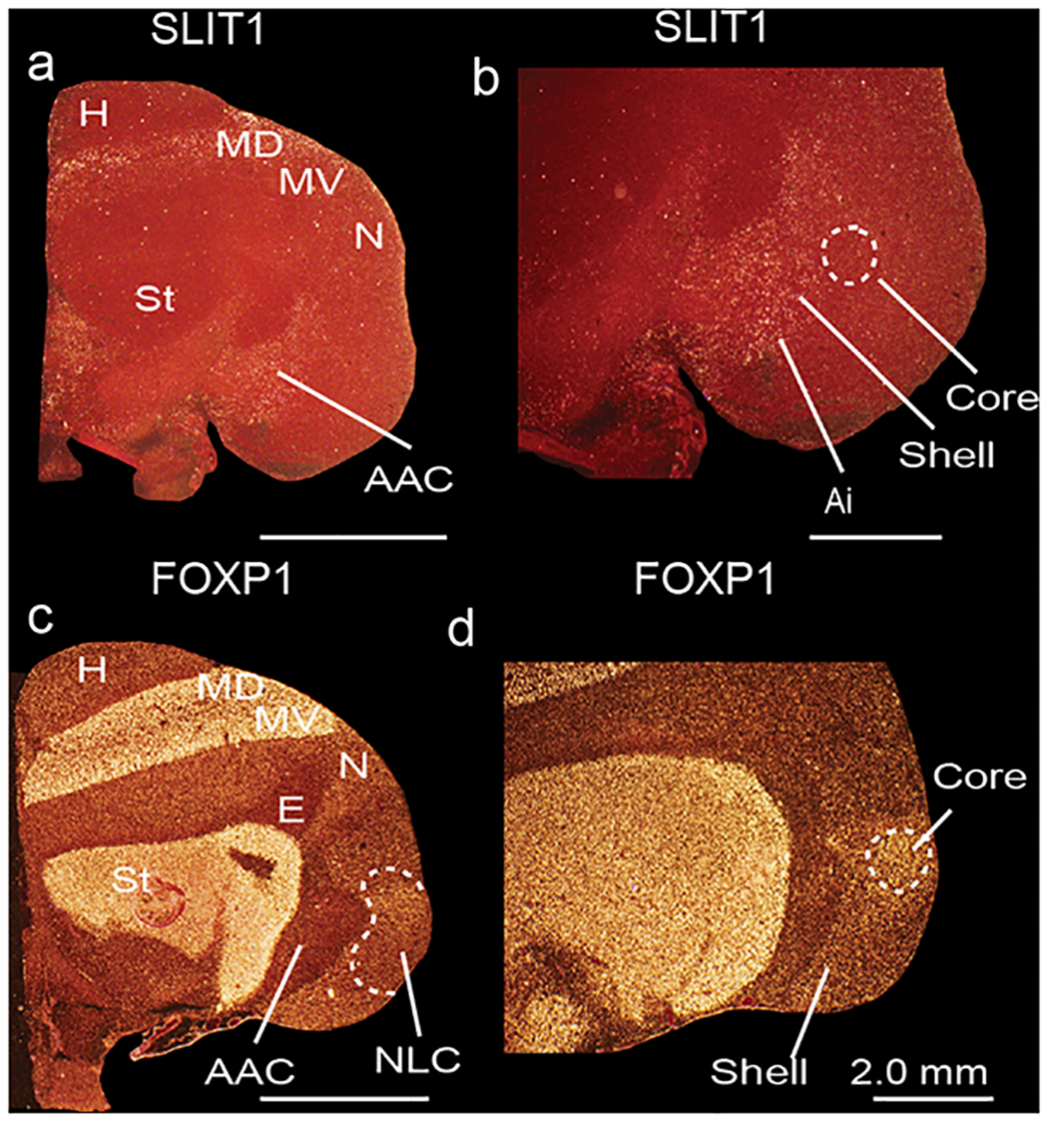
Darkfield photomicrographs showing specialized *SLIT1* and *FOXP1* mRNA expression in budgerigar song nuclei. (**a-b**) *SLIT1* mRNA is downregulated in the AAC core (dashed white lines), but not in the AAC shell relative to the surrounding arcopallium. (**c-d**) *FOXP1* mRNA is upregulated in the NLC core (dashed white lines) and part of the shell relative to the surrounding nidopallium. Sections are in the coronal plane; medial is to the left, dorsal is top. *FOXP1* images adapted from Feenders et al. [35]. See list for Abbreviations.

**Fig 8:**
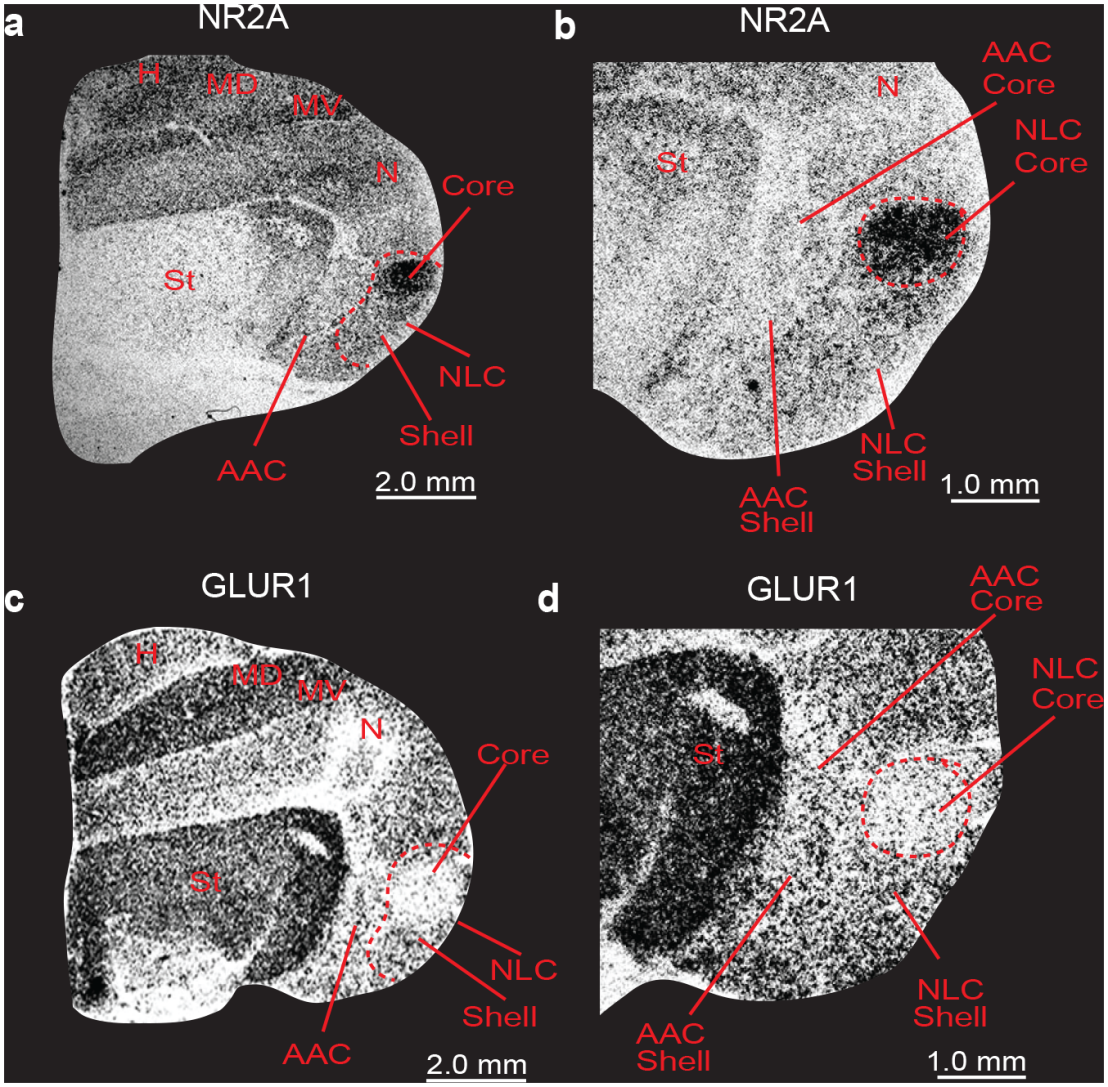
Brightfield photomicrographs of autoradiographs of *in situ* hybridizations showing specialized *NR2A* and *GluR1* mRNA expression in budgerigar song nuclei. (**a-b**) *NR2A* mRNA is upregulated in the NLC core (dashed red lines in b) relative to the surrounding nidopallium. (**c-d**) *GluR1* mRNA is downregulated in the NLC core (dashed red line in d) relative to the surrounding nidopallium. Sections are in the coronal plane; medial is to the left, dorsal is top. Sections are adjacent to those in Wada et al. [56]. See list for Abbreviations.

**Fig 9:**
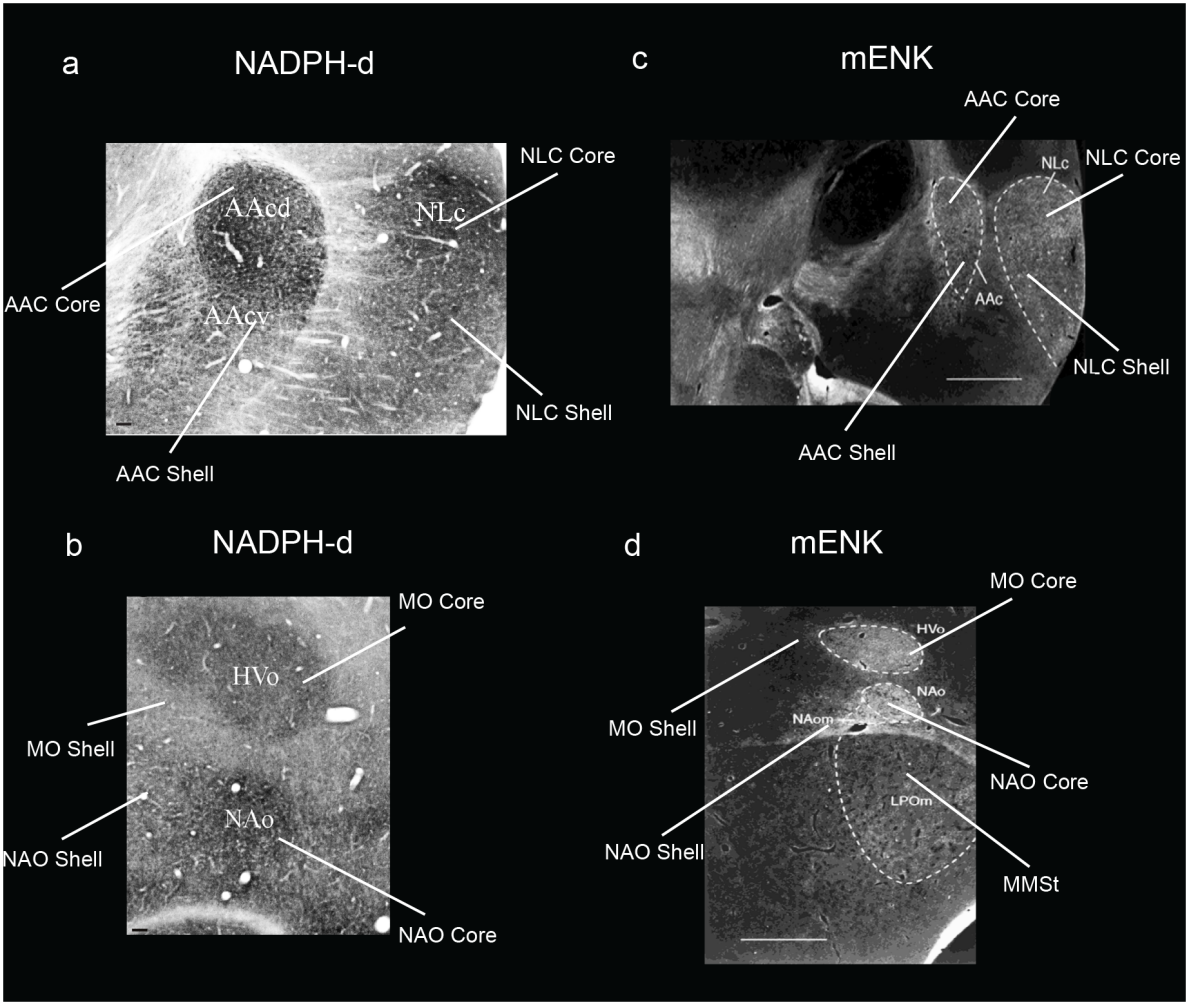
Brightfield and darkfield photomicrographs showing specialized NADPH-d and mENK staining in budgerigar song nuclei. (**a-b**) Brightfield photomicrographs showing higher NADPH-d staining in the NLC, AAC, MO, and NAO cores, and moderate specialized expression in the NLC, AAC, and MO shells. (**c-d**) Darkfield photomicrographs showing higher mENK staining in the NLC, AAC, MO, and NAO cores, and more moderate levels in the shells. Figures adapted from Brauth et al. [57] and Durand et al. [58], and reproduced with permission from S.E. Brauth and S.E. Durand. Abbreviations inside the images were based on an older nomenclature at the time. Sections are in the coronal plane; medial is to the left, dorsal is top. Scale bars = 100 μm in **a**—**b**, and 1mm in **c**—**d**.

**Fig 10:**
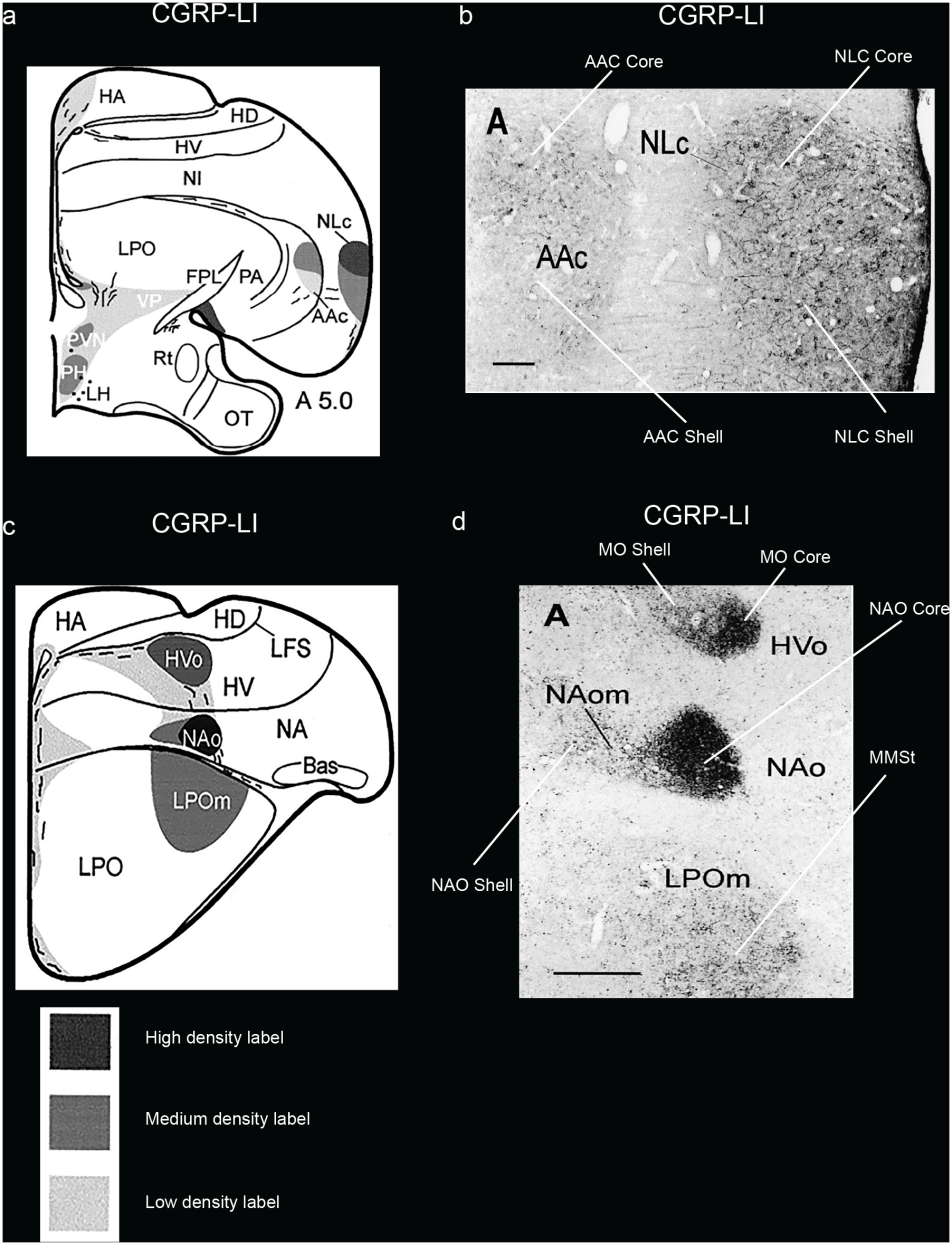
Brightfield photomicrographs showing specialized CGRP-LI staining in budgerigar song nuclei. (**a**) Camera lucida drawing summarizing the staining density of labeled fibers (key below indicates shading intensity) in the NLC and AAC song nuclei; (**b**) Photomicrographs showing higher CGRP-LI fiber and cell body staining in the NLC and AAC core and shell regions; (**c**) Camera lucida drawing summarizing the staining density of labeled fibers in the anterior song nuclei; (**d**) Photomicrographs showing higher CGRP-LI fibers in the MO and NAO cores. Figures adapted from Durand et al. [59], and reproduced with permission from S.E. Durand. Abbreviations inside the images were based on an older nomenclature at the time. Sections are in the coronal plane; medial is to the left, dorsal is top. Scale bars = 500 μm in **b** and **d**.

*SLIT1* is an axon guidance molecule that we had recently found was downregulated in songbird RA, hummingbird VA, and budgerigar dorsal AAC (AACd) [37,39]. We found here that the budgerigar region with *SLIT1* downregulation corresponds to our *PVALB*-defined AAC core, and that *SLIT1* expression in the *PVALB*-labeled AAC shell was not different relative to the surrounding arcopallium (Fig 7a and 7b). The *FOXP1* transcription factor, a co-factor of *FOXP2* that is required for speech and song acquisition in humans and songbirds [53], was reported to be differentially expressed in the NLC analog of vocal learners [35,54]; we found here that it is expressed in a gradient of higher to lower expression from the NLC core to the shell, but only for the part of the shell that corresponds to the vocalizing-driven song nucleus (Fig 7c and 7d). Interestingly, *FOXP1* has a ring of lower expression around the MMSt song nucleus, but this is thought to be a non-vocal motor part of the striatum around MMSt [35,55]. *NR2A* glutamate receptor upregulation (Fig 8a and 8b) and *GLUR1* downregulation in NLC [56] (Fig 8c and 8d) corresponds to our *PVALB*-defined NLC core. The NADPH-d enzyme pattern [57] resembles the *PVALB* pattern, with the exception that the NAO shell shows some specialized expression as well (Fig 9a and 9c). Methionine Enkephalin (mENK) fiber staining [58] also shows a similar pattern (Fig 9b and 9d). The CGRP-LI (Calcitonin gene-related peptide-like immunoreactivity) fiber staining [59] shows a diverse pattern, with comparable high density of cell body and fiber labeling in the NLC and AAC cores and shells, and barely any in the surrounding motor areas (Fig 10a and 10b); and a much higher density of fiber labeling than cell body labeling in the MO and NAO cores, less in the shells, and very little in the surrounding motor areas (Fig 10c and 10d). Matsunaga et al. [60] found higher androgen receptor (*AR*) expression in what we define here as both NLC, AAC, and NAO core and shells, and only the MO core (Table 1). Roberts et al. [61] found higher density of tyrosine hydroxylase (TH) fiber staining in the ventral part of NLC, which appears to correspond here to the *PVALB*-defined NLC shell and the motor part around the NLC shell compared to the NLC core where TH fibers were sparse (Table 1).

In songbirds and hummingbirds, we note that the song nuclei patterns of *SLIT1* [39], *FOXP1* [35,54], *NR2A* [56], and *GLUR1* [56] are similar to the core regions of the analogous nuclei in budgerigars. Overall, these results indicate that in budgerigars, the core and shell song regions, and the adjacent motor regions, have diverse patterns of specialized gene expression, and that the cores share more similar gene expression profiles with songbird and hummingbird song nuclei.

### Core and shell song regions show differences in connectivity

To investigate whether the gene expression-defined core and shell regions of the budgerigar song nuclei have similar or different neural connectivity, we collected and analyzed published and unpublished neural tracing data of male budgerigars, consisting of several thousand brain sections and performed side-by-side comparisons with the serial brain sections processed for *PVALB*, *SLIT1*, *FOXP1*, and vocalizing-driven IEG expression [24,31–34,38,45,54] (see methods, Table 2). Broadly, we noted that what had been called NLC in the prior connectivity studies consisted of both core and shell NLC regions defined by the gene expression profiles here (Table 3). What had been called dorsal AAC (AACd) corresponds to our AAC core; what had been called ventral AAC (AACv) contains part of our AAC shell. The previously named NLs overlying the NLC lies within the motor activated part outside of our NLC shell. What had been called NAO and MO corresponds only to our NAO and MO cores respectively (Table 3). The NAO and MO shell regions in previous connectivity studies were assumed to not belong to the song nuclei, which is understandable considering that they did not readily look conspicuous in Nissl staining. The regions identified in the connectivity studies were not tested functionally with vocal behavior, but they were tested with vocalizing-driven IEG expression [24], and when compared, the connectivity of the shells had some significant differences to the core nuclei. We present specific examples of one posterior and one anterior song nucleus below.

**Table 2:** Summary of neural connectivity findings.

Injection site	n =	AAC core	AAC shell	NLC core	NLC shell	MO core	MO shell	NAOcore	NAO shell
AAC core	3	+++	+o	ooo		+++o	+	++	
AAC shell	1		+++		ooo	++o			++oo
NLC core	1	+++		+++				o	
NLC shell	4		+++		+++	?	?	?	?
MO core	3	+		++		+++	++o	+++	+
MO shell	3		+		+	+o	+++	+o	+++o
NAO core	4	oo		++		+++	++	+++	+
NAO shell	4		+o		+		+oo		+++

**Fig 11:**
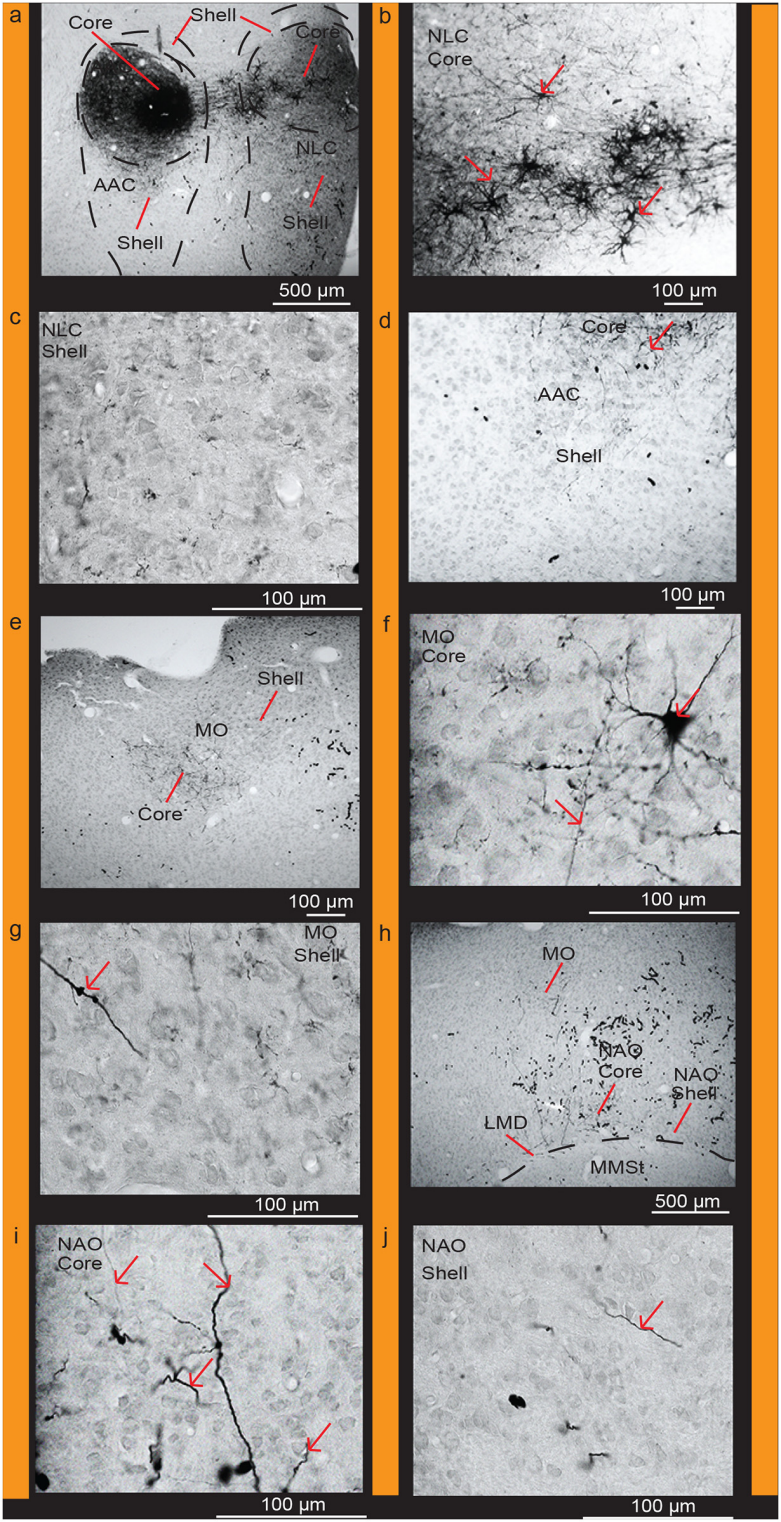
Connections of the AAC core. (**a**) Targeted injection of biocytin in the AAC core region; (**b**) High power view of labeled cells and fibers in the NLC core; (**c**) Very few cells and fibers in the NLC shell; (**d**) Very sparse fibers in the AAC shell; (**e**) Densely labeled fibers in the MO core; (**f**) High power view of labeled cells and fibers in the MO core; (**g**) High power view of a labeled fiber in the MO shell; (**h**) Labeled fibers in the NAO core; (**i**) High power view of labeled fibers in the NAO core; (**j**) High power view of a labeled fiber in the NAO shell. Red arrows indicate labeled cells and fibers. Black worm like structures are blood vessels that were not completely perfused. Sections are in the coronal plane; medial is to the left, dorsal is top. See list for Abbreviations.

**Fig 12:**
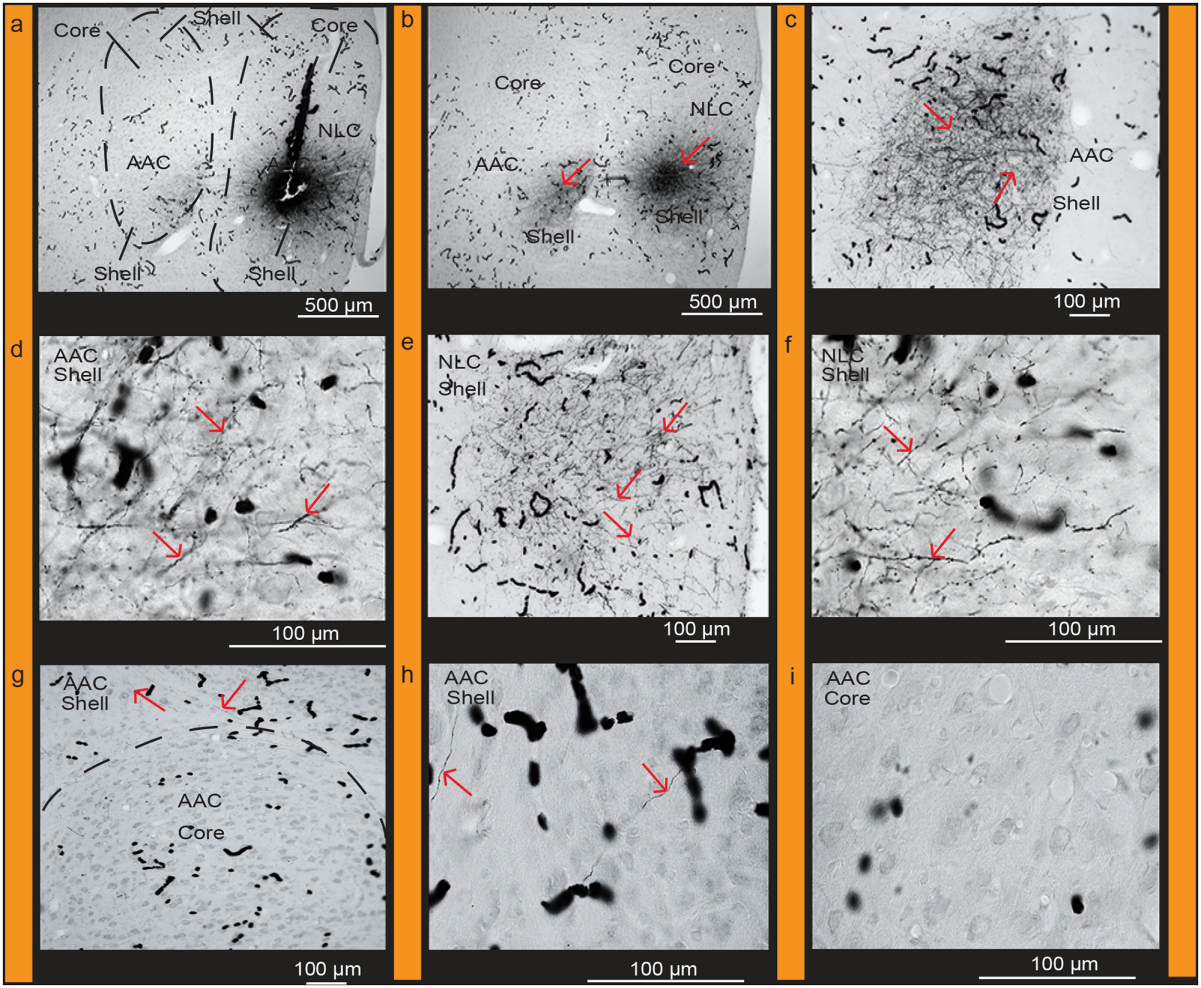
Connections of the NLC shell. (**a**) Targeted injections of biocytin in the NLC shell region. (**b**) Densely labeled fibers in the AAC shell but absent in the core; (**c**) Clusters of labeled fibers in the AAC shell; (**d**) High power view of labeled fibers in the AAC shell; (**e**) Dense clusters of labeled fibers in the NLC shell; (**f**) High power view of labeled fibers in the NLC shell; (**g**) Labeled fibers present in the AAC shell immediately dorsal to the core; (**h**) High power view of labeled fibers in the AAC shell; (**i**) Absence of labeled fibers in the AAC core. Red arrows indicate labeled cells and fibers. Black worm like structures are blood vessels that were not completely perfused. Sections are in the coronal plane; medial is to the left, dorsal is top. See list for Abbreviations.

**Fig 13:**
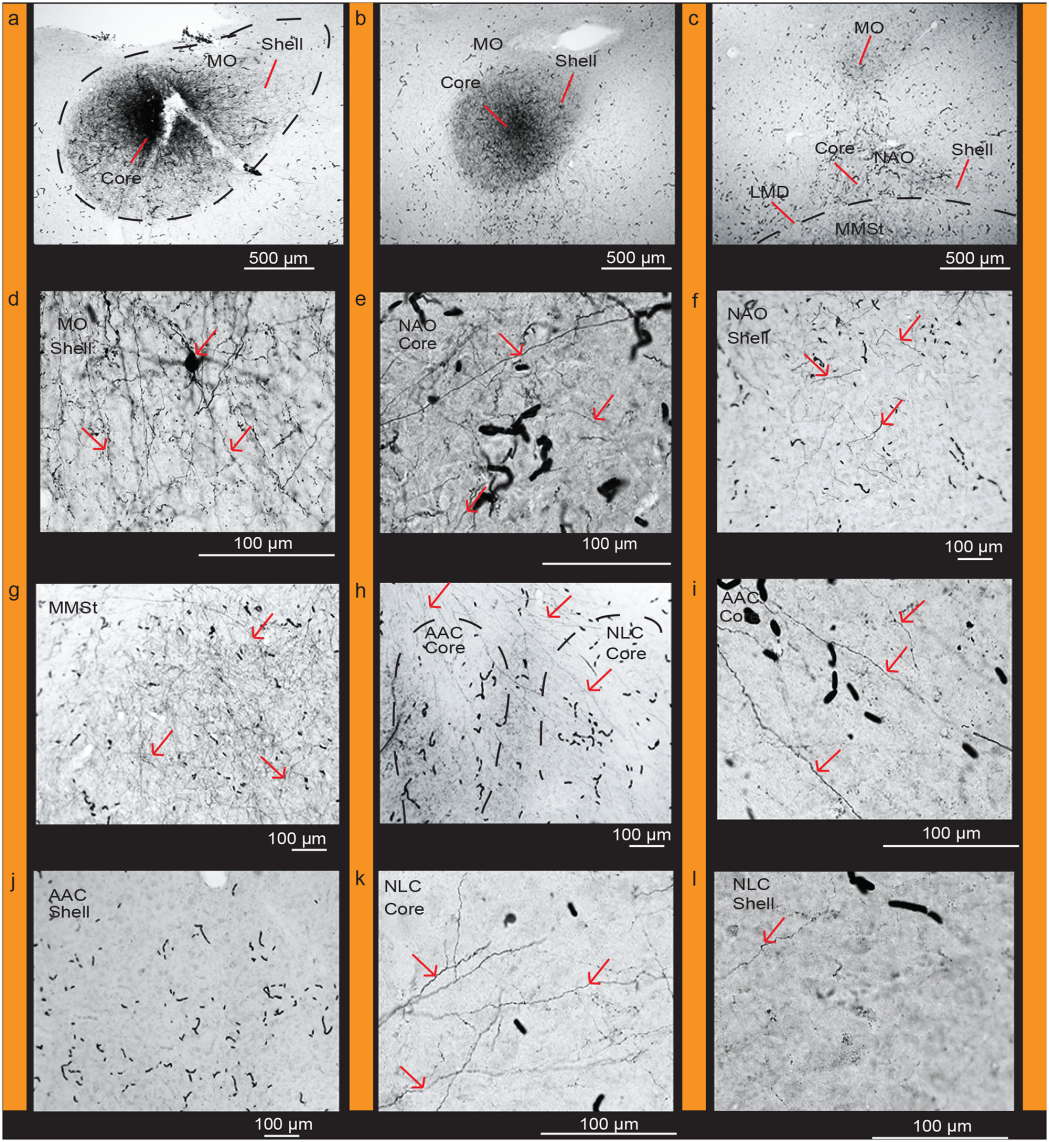
Connections of the MO core. (**a**) Targeted injection of biocytin in the MO core; (**b**) Densely labeled fibers in adjacent parts of the MO core and shell; (**c**) Densely labeled fibers within the MO core, NAO core, and in MMSt, and sparse fibers in the NAO shell; (**d**) Densely labeled fibers and cells in the MO shell; (**e**) High power view of labeled fibers in the NAO core; (**f**) Sparse labeled fibers present in the NAO shell; (**g**) Dense clusters of labeled fibers in MMSt; (**h**) Clusters of fibers projecting to the NLC core and AAC core; (**i**) High power view of labeled fibers in the AAC core; (**j**) Absence of fibers in the AAC shell (**k**) Clusters of labeled fibers in the NLC core; (**l**) Sparse fibers present in the NLC shell. Red arrows indicate labeled cells and fibers. Black worm like structures are blood vessels that were not completely perfused. Sections are in the coronal plane; medial is to the left, dorsal is top. See list for Abbreviations.

**Fig 14:**
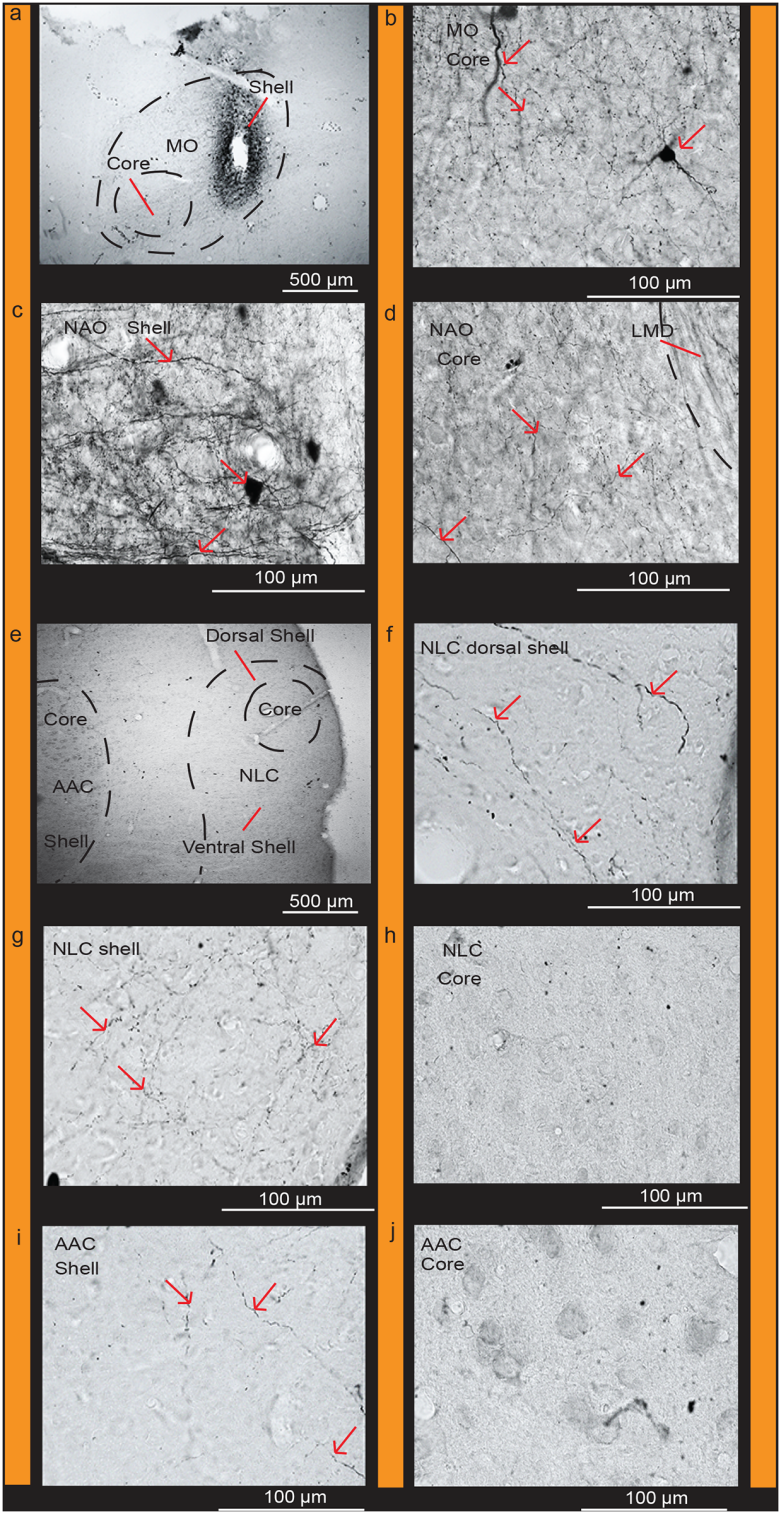
Connections of the MO shell. (**a**) Targeted injection of BDA in the MO shell; (**b**) High power view of labeled fibers and cells in the MO core; (**c**) High power view of densely labeled cells and fibers in the NAO shell; (**d**) Sparse labeled fibers present in the NAO core; (**e**) Labeled fibers are present in the dorsal and ventral NLC shell; (**f**) High power view of labeled fibers present in the dorsal region of the NLC shell; (**g**) Labeled fibers present in the ventral region of the NLC shell; (**h**) No labeled fibers or cells visible in the NLC core; (**i**) Labeled fibers in the AAC shell; (**j**) No labeled fibers or cells visible in the AAC core. Red arrows indicate labeled cells and fibers. Black worm like structures are blood vessels that were not completely perfused. Sections are in the coronal plane; medial is to the left, dorsal is top. See list for Abbreviations.

Sample sizes are number of animals with injections. Listed are qualitative analyses of relative degree of staining of fibers (+) and/or labeled cells (o) present in the song nucleus; no value indicates that no labeled fibers or cells were visualized in these areas. See Figs 11–14 for representative examples of tracer injection sites and spread of tracer and results for explanation of findings and sources of experiments.

**Table 3:** List of abbreviated names of song nuclei used in describing the parrot song system by various studies interpreted relative to our study.

Paton et al. 1981	Striedter, 1994	Durand et al. 1997	Jarvis et al. 2000	Reiner et al 2004, Jarvis et al 2005	Present study
HVc	NLc	NLc	NLc	NLC	NLC core
	NLc	NLc	NLc	NLC	NLC shell
RA	AAc	AAcd	AAcd	AAcd	AAC core
	AAc	AAcv*	AAcv*	AAcv	AAC shell
	HVo	HVo	HVo core*	MO core	MO core
			HVo surround*	MO surround	MO shell
MAN	NAo	NAo	NAo core*	NAO core	NAO core
MAN	NAs*	NAom*	NAo surround*	NAO surround	NAO shell

* NAs defined in Striedter [32] is a small portion of what we find as the NAO shell, in which authors said was analogous to songbird LMAN. NAom defined in Durand et al. [34] is another small portion of what we define as NAO shell. NAo core and surround and HVo core and surround in Jarvis et al. [24] was together called the NAo and HVo complex. AAcv in Durand et al. [34] and Jarvis et al. [24] comprised the ventral part of the AAC shell described here.

When analyzed in the context of our differential and functionally-defined (vocalizing-driven) gene expression profiles and Nissl-defined patterns, we found that targeted biocytin injections into the AAC core (Fig 11a) with very little leakage in the shell retrogradely labeled a high density of cell bodies and some anterogradely labeled fibers within the *PVALB*-defined (from strongest to weakest) NLC core, MO core, and NAO core relative to the shell regions (Fig 11b–11j; Table 2). We did not observe fiber labeling in the MMSt region (Fig 11h) consistent with prior studies [34]. Targeted biocytin injections within the AAC shell labeled cell bodies and some fibers in the NLC shell and NAO shell, and mostly fibers in the MO core (Figs 6b, 6c and 7 of Durand et al. [34]; Table 2). Conversely, targeted biocytin injections within the NLC shell (Fig 12a) with little to no leakage in the core (Fig 12a and 12b) labeled a high density of fibers in the AAC shell and NLC shell (Fig 12c–12h; Table 2), supporting the shell-to-shell connectivity. We did not observe labeled fibers within the AAC core (Fig 12i). These findings are consistent with those of Striedter [32], who showed topographic connectivity between the AAC and NLC song nuclei, which we now define as the AAC and NLC cores and shells.

Targeted biocytin injections in the *PVALB*-defined MO core (Fig 13a and 13b) labeled a higher density of fibers within (from strongest to weakest) the NAO core (Fig 13c and 13e), NLC core (Fig 13h and 13k), and AAC core (Fig 13h and 13i) relative to their shells (Fig 13f, 13j and 13l). There were also fiber projections and filled cell bodies in the MO shell (Fig 13c and 13d) and MMSt of the striatum (Fig 13g). Targeted BDA (biotinylated dextran amine) injections within the MO shell (Fig 14a) with little to no leakage in the core, labeled fibers and some cell bodies within the MO core (Fig 14b) confirming the local connectivity finding. However, there were greater amounts of labeled fibers and some cell bodies in the NAO shell (Fig 14c), than in the NAO core (Fig 14d), and fibers only in the NLC and AAC shells (Fig 14e–14j; Table 2). These findings suggest a strong core-to-core connectivity of the MO with the other core song nuclei, strong shell-to-shell connectivity of MO with the other shell song nuclei, and a weaker local MO and NAO shell with core connectivity.

**Fig 15:**
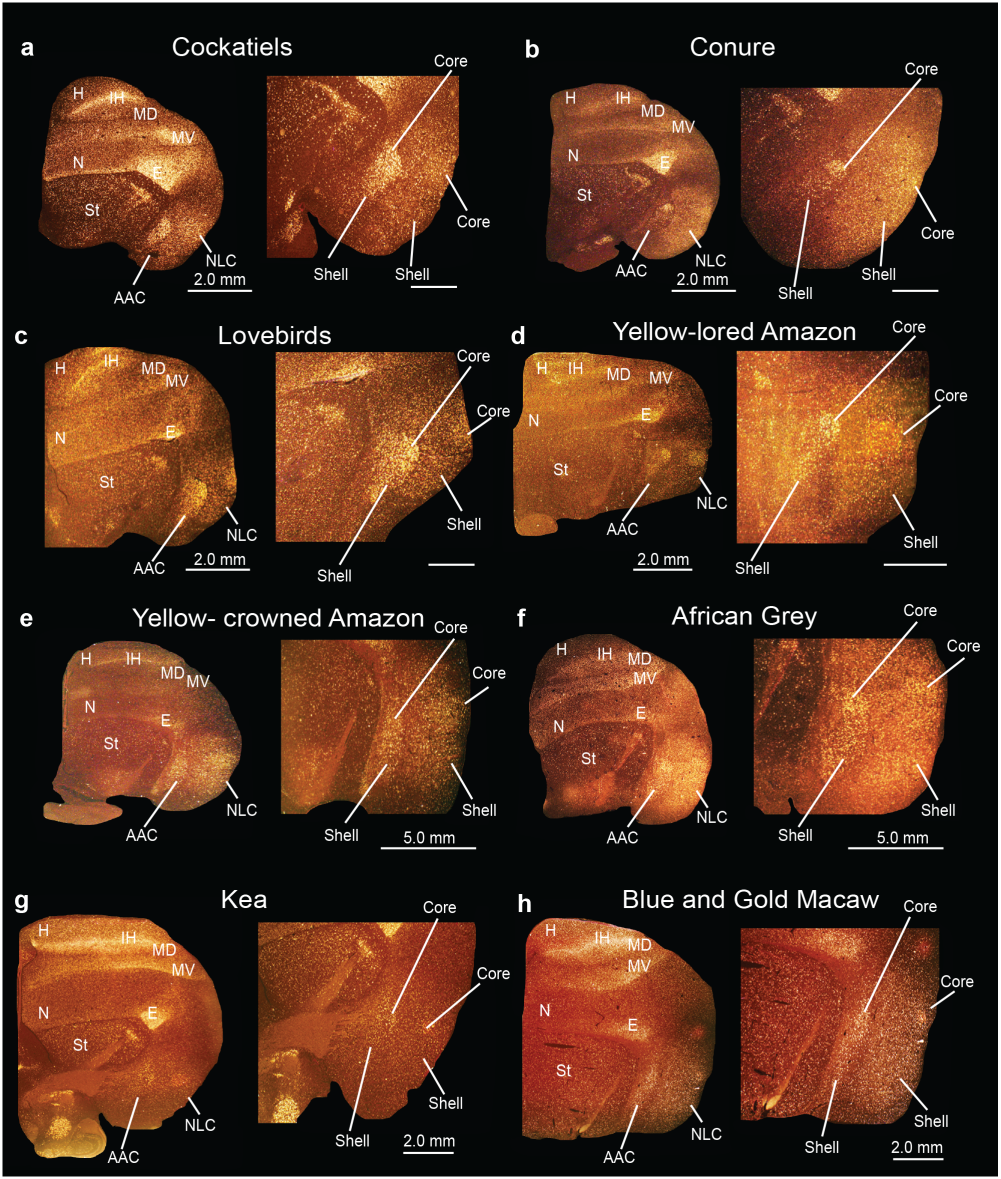
Posterior song nuclei in different parrot species. (**a-h**) Low and high power views of darkfield images showing *PVALB* mRNA expression in the core and shell regions of NLC and AAC of eight parrot species. Silver grains in white; red-brown is cresyl violet staining. Sections are in the coronal plane; medial is to the left, dorsal is top. Brain sections are not to scale, due to large size differences between species. See list for Abbreviations.

Available published data for the remaining song nuclei also support parallel core and shell systems. For example, injections of dual anterograde+retrograde fluorescent dextran amines targeted to the NAO core (i.e. NAom) by Durand et al [34], labeled cells mostly confined to what we define as the AAC core, but with a smaller minority in the shell (Table 2; and Fig 15A and 15B of that study). Injections of fluorescent dextran amines in the NAO shell labeled cells and fibers in the AAC shell (Table 2; Fig 15A and 15D of that study).

Taken together, these findings indicate that the core regions of NLC, AAC, MO, and NAO are primarily interconnected to each other whereas the shell regions are primarily interconnected to each other. There are sparser connections between cores and shells within and among each song nucleus.

### Identification of song nuclei specializations in other parrot species

**Fig 16:**
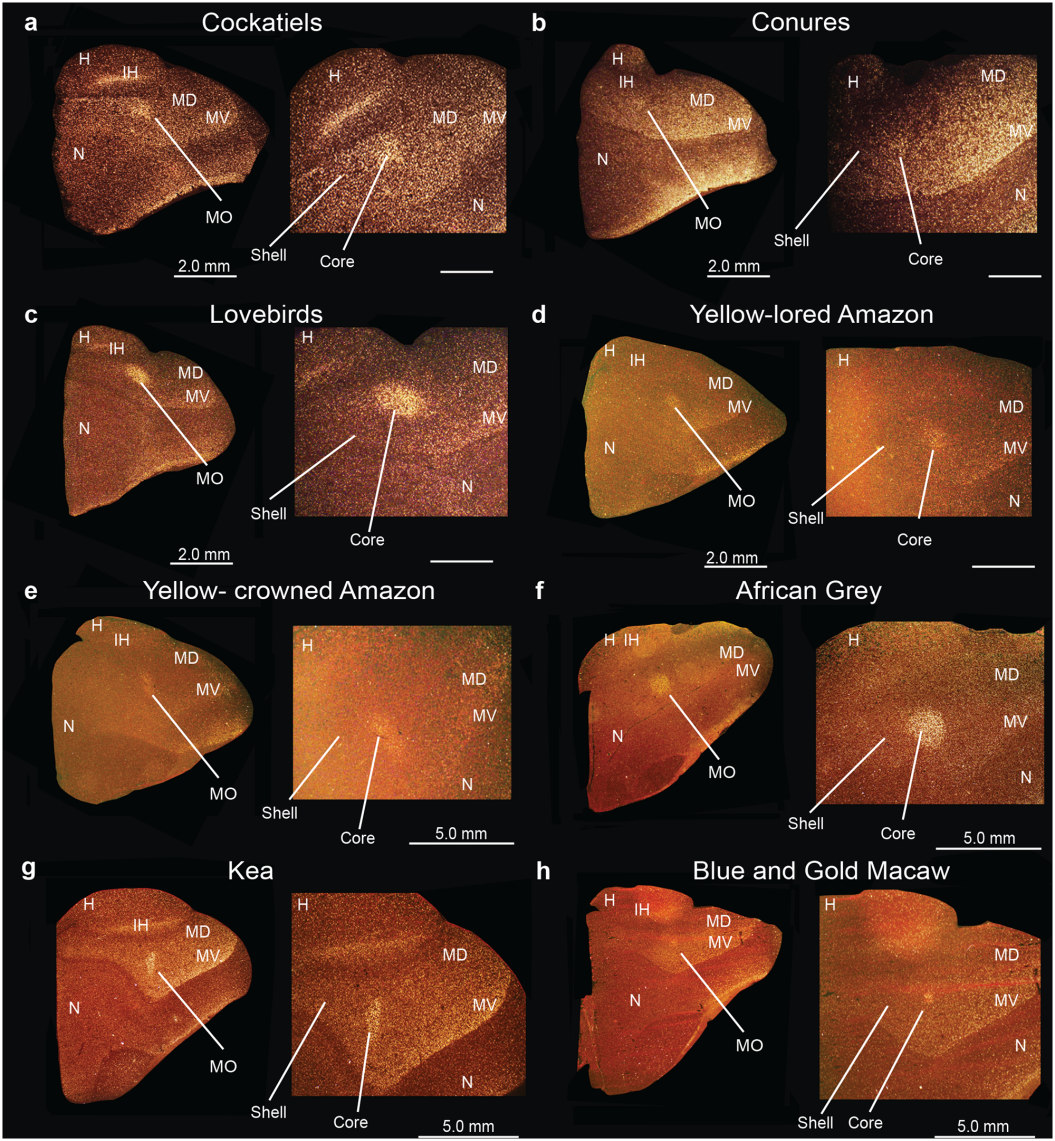
Anterior song nucleus MO in different parrot species. (**a-h**) Low and high power views of darkfield images showing *PVALB* mRNA expression in the core region of the MO song nucleus in eight parrot species. Silver grains is white; red-brown is cresyl violet staining. Sections are in the coronal plane; medial is to the left, dorsal is top. Brain sections are not to scale, due to large size differences between species. See list for Abbreviations.

**Fig 17:**
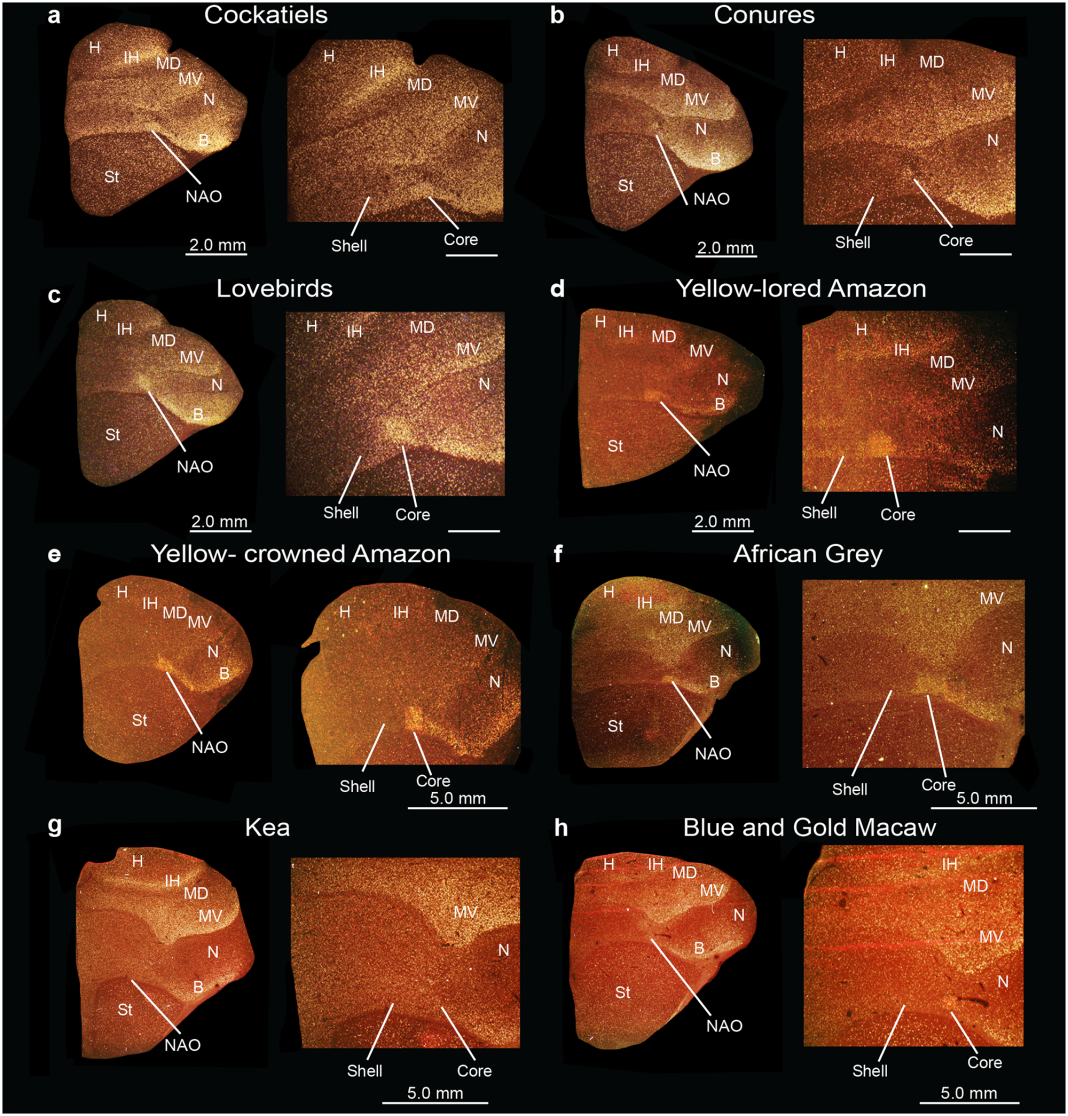
Anterior song nucleus NAO in different parrot species. **(a-h)** Low and high power views of darkfield images showing *PVALB* mRNA expression in the core region of the NAO song nucleus in eight parrot species. Silver grains is white; red-brown is cresyl violet staining. Brain sections are not to scale, due to large size differences between species. Sections are in the coronal plane; medial is to the left, dorsal is top. See list for Abbreviations.

We wondered if the core and shell song nuclei organization was unique to budgerigars, or to parrots generally. Using *PVALB* mRNA expression, we discovered the presence of similarly labeled song nuclei-like regions in eight other parrot species, namely the yellow lored Amazon (*Amazona xantholora*), yellow crowned Amazon (*Amazona ochrocephala*), peach-faced lovebird (*Agapornis roseicollis*), African Grey parrot (*Psittacus erithacus*), the peach-fronted conure (*Aratinga aurea*), and the blue and gold macaw (*Ara ararauna*), all from the Psittacidae Family, the cockatiel (*Nymphicus hollandicus*) from the Cacatuidae Family, and the kea (*Nestor notabilis*) from the Nestoridae Family (Figs 15–17) [62]. Similar to budgerigars, we distinguished *PVALB*-defined NLC and AAC core (high expression) and shell (moderate expression) regions (Fig 15), and only the MO and the NAO cores (Figs 16 and 17). The kea, considered the most distantly related of the group and representing the most basal split with the other species [8], contained distinct *PVALB-* and Nissl-defined NLC, ACC, and MO core regions similar to other species (Figs 15g and 16g). However, the kea *PVALB*- defined MO core was rectangular in shape (Fig 16g) in contrast to the more typical oval shape found in other species, and the kea did not possess a detectable NAO core with high *PVALB* expression that we could identify (Fig 17g) in serial sections, although a tiny core region was distinguishable with Nissl staining. We also noted that differential expression of *PVALB* in the kea NLC and AAC shell regions (presumably including motor areas) was barely detectable relative to the surrounding brain regions (Fig 15g).

**Fig 18:**
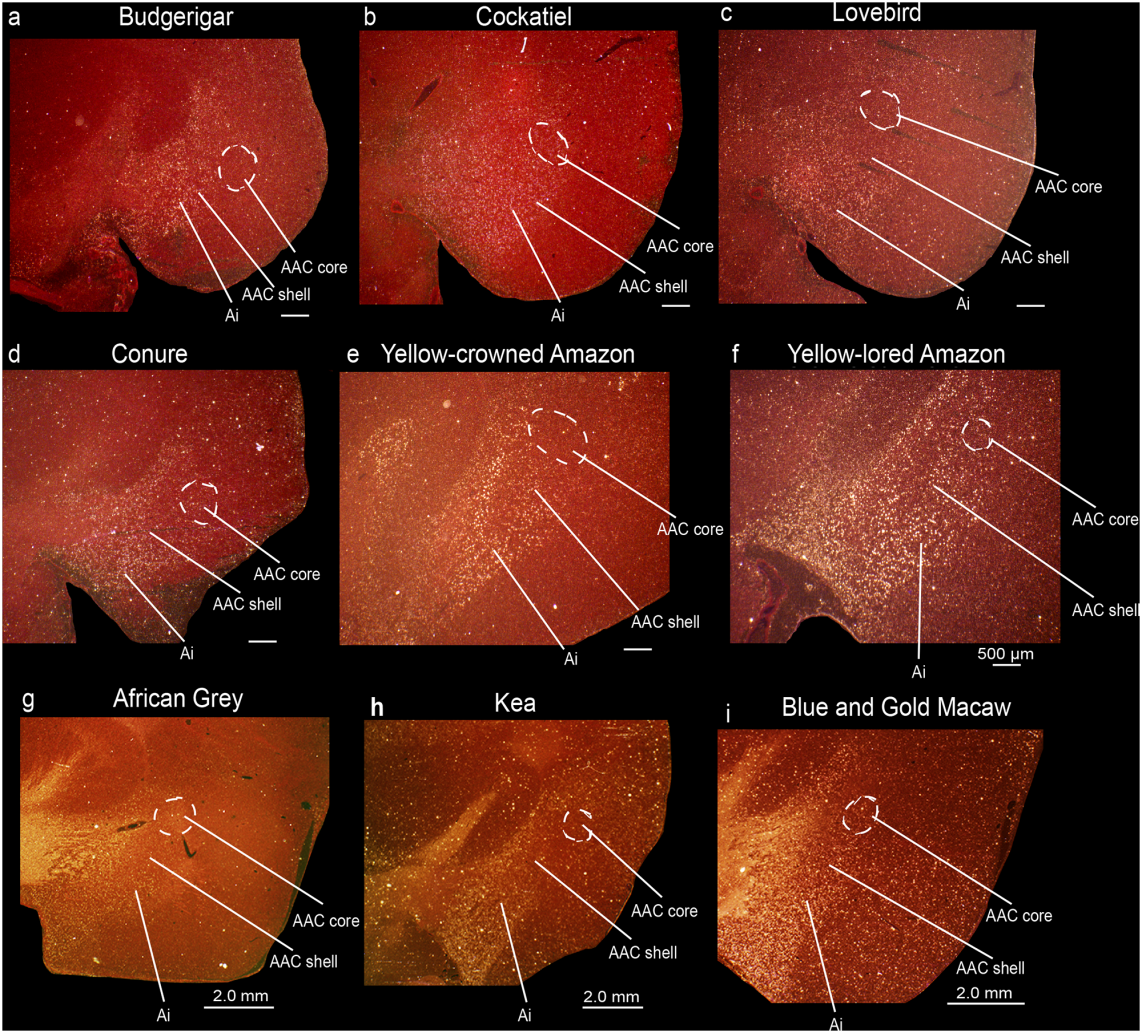
Darkfield images of *SLIT1* mRNA expression in the core and shell regions of the AAC song nucleus in nine parrot species. (**a-b**) *SLIT1* mRNA expression is downregulated (low expression) in the AAC core (dashed white lines) but not AAC shell region relative to the surrounding arcopallium in budgerigars and cockatiels. (**c-i**) *SLIT1* mRNA expression is downregulated in the AAC core region (dashed white lines) and shows intermediate levels in the AAC shell region relative to higher levels in the surrounding arcopallium in peach-faced lovebirds, peach-fronted conures, yellow crowned Amazon, yellow lored Amazon parrots, African Grey, kea, and blue and gold macaw. Sections are in the coronal plane; medial is to the right, dorsal is top. Scale bar = 500 μm, 2.0 mm. See list for Abbreviations.

Different species had variation in specialized *SLIT1* expression in the AAC song nucleus. Similar to budgerigars, *SLIT1* mRNA was downregulated in the AAC core relative to the AAC shell and the surrounding arcopallium in cockatiels (Fig 18a and 18b). However, in the peach-faced lovebird, peach-fronted conure, the two Amazon species, the African Grey, the kea, and in the blue and gold macaw not only was *SLIT1* downregulated in the AAC core region, but it was expressed at noticeably lower levels in the AAC shell relative to the surrounding arcopallium (Fig 18c–18i). This resulted in two gene expression boundaries in the *in situ* hybridizations, one for the core and another one for the shell. The boundaries of the shell appeared to coincide with the *PVALB*-defined shell boundaries in the adjacent sections. We surmise that the *SLIT1* specialized shell expression in these seven parrot species could possibly contain both the song nucleus and adjacent motor areas.

### Core and shell cross-section area ratios show species differences

**Fig 19:**
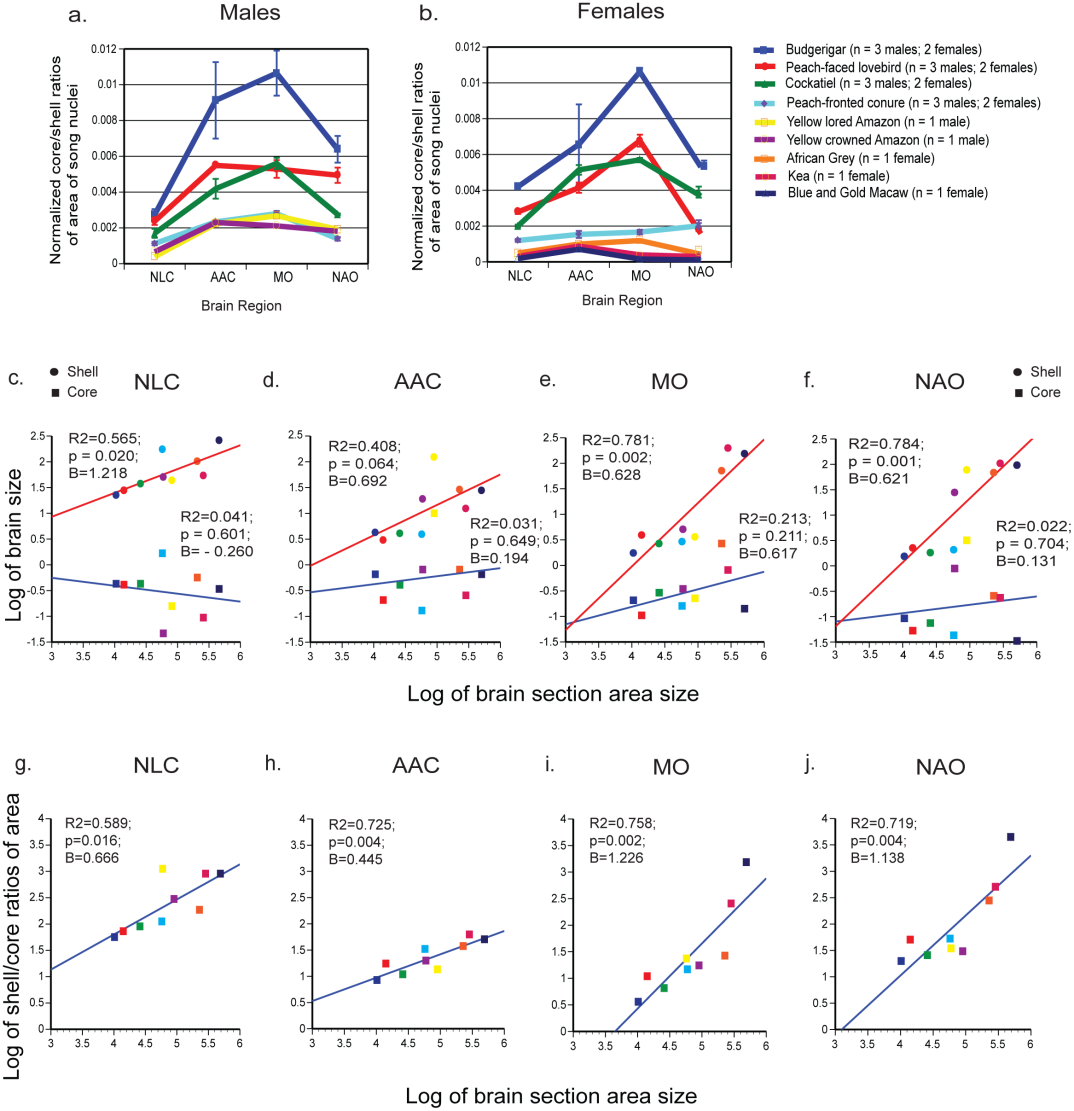
Differences in core and shell song nuclei area sizes across species. (**a**) Ratios of core and shell areas of song nuclei in males; (**b**) Ratios of core and shell areas of song nuclei in females. Values were normalized to brain section size, from the same brain sections that core and shell measurements were taken from. Error bars are S.E.M., in cases where we had n > 1 animal. (**c-f**) Regression log scale plots of core and shell nuclei area sizes relative to brain section area size, to test for presence of isometric (slope B ~1; p < 0.05) or allometric (slope B >1 or <1; p < 0.05) scaling. (**g-j**) Regression log scale plots of shell:core (inverse of core:shell in b) nuclei area size ratios relative to brain section area size, to determine if the ratio is related to the brain section size.

We noted considerable relative size differences in the core and shell regions among species (Figs 15–17). Thus, we analyzed the area ratios of the core and shell regions of NLC, AAC, MO and NAO in all nine species by sampling serially adjacent brain sections (n = 3–4 per animal) where the core region was the largest (in the center part of the nucleus) using *PVALB* and Nissl-defined boundaries. We separately quantified males (Fig 19a) and females (Fig 19b), but did not have a large enough sample size of each sex for each species to reliably determine if there were species differences between the sexes. We found that budgerigars and cockatiels had the highest core to shell area ratios overall (i.e. cores were larger), followed by lovebird, conures, and the two Amazons, whereas the African Grey, kea and the blue and gold macaw were among the lowest (i.e. cores were smaller; Fig 19a and 19b). The nidopallial song nuclei NLC and NAO generally had the smallest core to shell area ratios, the arcopallium song nucleus AAC intermediate levels, and the mesopallium song nucleus MO the highest ratios (i.e. core was larger; Fig 19a and 19b). Females of some of the larger brained species (conure, kea, and macaw) did not follow this same pattern (Fig 19b).

To test for isometric versus allometric scaling of core and shell sizes relative to brain size, we measured brain section area from the same sections as a proxy for brain size; we did not have sufficient brain sections to measure volume. We found that the core and shell regions measured did not increase in size isometrically (e.g. not the same slopes in Fig 19c–19f). Rather, the shells had a significant log-linear relationship with their brain section size, which was stronger for the anterior song nuclei, whereas the cores did not. If anything, the NLC core had a trend for the opposite relationship (Fig 19c). Except for NLC, the slopes of shell values were less than 1 (B = ~0.6), meaning a negative allometric relationship with brain section size. Despite the cores not having a scaling relationship, there was a strong log-linear relationship of shell:core ratios with brain section size (Fig 19g–19j). This relationship was negatively allometric (B << 1) for the posterior nuclei NLC and AAC, and weakly positively allometric if at all (B > or ~1) for the anterior nuclei MO and NAO. Only the relationship of AAC with brain section size become stronger, indicating that except for this nucleus, most of the scaling relationship with brain size is due to the shells changing size.

Overall, these findings demonstrate a significant plasticity in relative sizes of song nuclei among parrot species, with the shell regions being larger in larger brain species that are also typically considered to have more advanced vocal and cognitive abilities. The core varies, but not with brain section size alone, indicative of another variable, perhaps behavior, that its size could be associated with.

## Discussion

**Fig 20:**
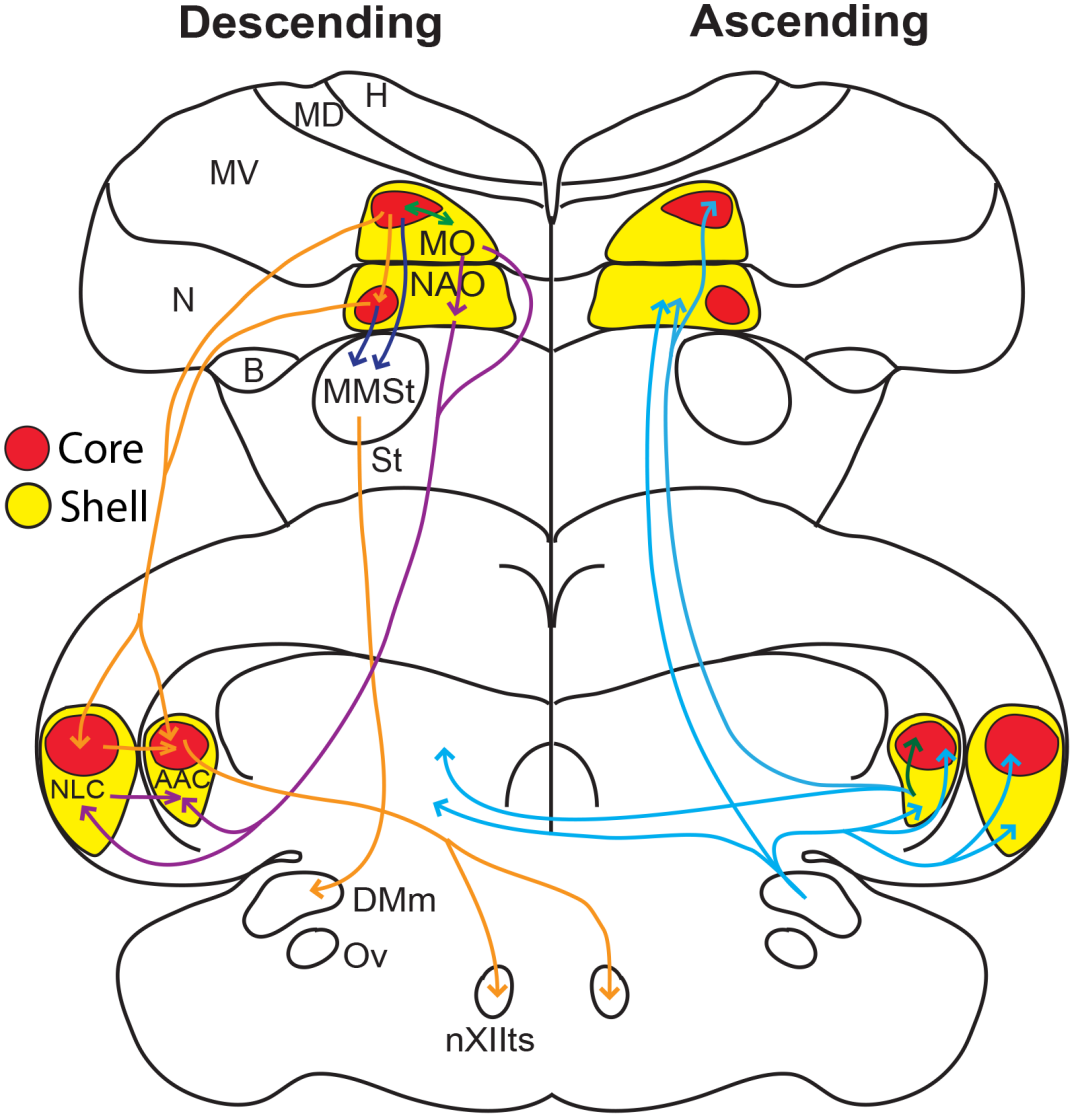
Schematic drawing summarizing the findings of this and other studies. Drawn are sections in the coronal plane through a budgerigar brain, showing the core (red shapes) and surrounding shell (yellow shapes) song nuclei. Arrows show descending core-to-core pathways (orange), descending shell-to-shell pathways (purple), descending connections to MMSt (dark blue), ascending bilateral song pathways (blue), and shell and core interactions within the same song nucleus (green). The connections are based on findings in this and prior studies of Durand et al. [34], Brauth et al. [31], Striedter [32], and Paton et al. [33].

Our study provides several advances in our understanding of the parrot song system. First, unlike other vocal learning bird lineages, the parrot song system has core and shell regions. Second, the *PVALB*-defined shell specializations include song and adjacent motor areas for both posterior song nuclei. Third, the core and shell regions show differences in neural connectivity, with stronger connections among the core regions and among the shell regions (summarized in Fig 20). Fourth, for both gene expression specializations and neural connectivity, the parrot core system is more similar to the song systems of songbirds and hummingbirds. Fifth, apparent *PVALB*-, *SLIT1*-, and Nissl-defined core and shell song nuclei exist in other parrot species besides the budgerigar, where there are relative size differences of the core and shell regions among species. Finally, the existence of song nuclei in the kea, but with some notable differences from the other species, demonstrates that song nuclei evolved early in parrots more than 29 million years ago before the kea split from the other parrot lineages (based on a dated genome-scale phylogenetic tree [8]), with subsequent divergences of their systems. Based on these results, we hypothesize that the parrot brain consists of a song system embedded within a song system. Below we discuss the implications of this hypothesis.

Our study helps clear up contradictions between past studies, in that different investigators had been studying the core, shell, and/or both without consistent recognition of the differences between them (Table 3). In the initial discovery of the budgerigar song system, Paton et al. [33] identified the posterior song pathway and called the nuclei by their songbird analogs HVC and RA, which we note in their figures appears to precisely correspond to our NLC core and AAC core respectively (Table 3). Paton et al. [33] also identified a large region that they named MAN similar to the comparable nucleus songbirds, which corresponds to our NAO core and shell. Striedter [32] renamed the posterior nuclei to NLC and AAC due to the belief that the parrot song system had evolved independently of songbirds, and expanded the regions to encompass most but not all of our shells. In the same study, Striedter [32] also identified our MO core (called HVo then) and NAO core, but did not consider them analogs of any songbird song nuclei, and instead considered a region he called NAs adjacent to the NAO core (part of our NAO shell) as the LMAN analog of songbirds (Table 3). Durand et al. [34] also identified the boundaries of NLC and AAC but did not distinguish the core and shell regions. Further, Durand claimed that NAom (a medial part of our NAO shell) adjacent to NAo (our core) is part of the parrot song system. Jarvis and Mello [24], using vocalizing-driven IEG expression, functionally identified the MO and NAO core and shells, calling them core and surround complexes, but did not recognize a core and shell for the NLC and AAC nuclei (Table 3). Since then, different studies have used either the Jarvis and Mello [24] or Durand et al. [34] naming systems without reconciling the two. In 2004, a new nomenclature for the avian brain was published that more closely matched their mammalian homologs [63,64]. The new nomenclature resulted in changes to the names (but not abbreviations, except for MO and MMSt), but it did not resolve the discrepancies. In the current study, authors from both groups reconciled the differences based on the findings of this study (Table 3).

We did not find gene expression specializations for the two smaller parrot cortical song nuclei, LAN (lateral nucleus of the anterior nidopallium) in the nidopallium and LAM (lateral nucleus of the anterior mesopallium) in the mesopallium (analogs of songbird NIf and Av) [24], except for distinct mENK labeled fibers and cells (Fig 12B of Durand et al. [58]). We note that variation in mENK expression among animals could be related to singing as it was later discovered that mENK expression levels in several songbird song nuclei are singing regulated [46].

One fundamental shared connection among the vocal learning birds is the parrot’s AAC core, songbird’s RA, and hummingbird’s VA specialized direct projection to the brainstem vocal lower motor neurons [34]; a difference is that the parrot AAC shell does not make this projection and instead projects to adjacent forebrain song nuclei (Fig 20). The direct projection to vocal motor neurons is thought to be critical for the evolution of vocal learning, as vocal non-learning species either do not have it or have a very sparse projection [3,6,65–68]. Given this interpretation, and the more rudimentary presence of the shell regions and absence of a detectable specialized NAO core region, but clear presence of other core regions in the kea, the “core song system” could represent a more “ancestral” state in the evolution of vocal learning in parrots that is similar with songbirds and hummingbirds, and the “shell song system” represent a more recently evolved state unique to parrots. If there were three independent gains of vocal learning, then parrots, songbirds, and hummingbirds would have each evolved the “core system” independently, with subsequent evolution of the shell system in only parrots. If there were only two independent gains, then the common ancestor of parrots and songbirds, and the ancestor of hummingbirds would have each evolved a core-like system independently, and thereafter suboscines and possibly New Zealand Wrens lost the core system and the vocal learning trait, whereas parrots went in the opposite direction and enhanced the trait with subsequent evolution of the “shell system”. In support of a possible loss, it was recently found that the suboscine Eastern phoebe (*Sayornis phoebe*) has a rudimentary RA (AAC-like) song nucleus not found in other bird species, such as quails [69], and at least another suboscine, a Bellbird (*Procnias contigas*), shows evidence for vocal learning [70] whereas multiple others do not [71]. We also note that different types of specializations in the hummingbird NLC (VLN) and NAO (VAN) analogs exist, which could also be due to subsequent changes after their song system first evolved. Future experiments will be necessary to resolve the two hypotheses.

The remarkable relative size differences between core and shell regions among parrot species could reflect functional and/or brain size differences. Parrot species generally considered to be more limited in their vocal imitation abilities, such as the budgerigar, peach-faced lovebirds, and cockatiels, have larger and conspicuous core nuclei relative to the shell nuclei, whereas those thought to have considerably more complex communication abilities such as the blue and gold macaws, peach-fronted conures, African Grey [15,17], yellow lored Amazon, and yellow crowned Amazon have noticeably smaller cores, and correspondingly larger shell regions. Consistent with this notion, recent behavioral studies by Walløe [72] have indicated that the peach-fronted conures show rapid vocal modification abilities compared to other species such as the budgerigars. We noted that except for the kea MO, the shapes of the song nuclei across species were very similar in serial sections, suggesting that shape differences are unlikely to confer differences in the relative sizes of the core and shell regions. Another possibility is that the differences in core and shell ratios are due to allometric scaling, where the shell could become disproportionally larger than the cores as the brain and body evolved to become larger. However, because we find that not all shell regions scale allometrically (i.e. NLC), the shell scaling of other song nuclei is negatively allometric, and the cores from the very same sections do not show significant scaling with brain section size, this suggests that the shell and core systems can be scaled somewhat independently of each other and from brain size. Further, allometric scaling for the shell also does not mean that larger brain regions (shells) are not involved in more complex aspects of vocal communication. The kea, also a larger brain parrot, does not show a robust shell specialization. Very little has been reported on kea vocal communication abilities, but one study has shown that there is geographic variation in their contact calls, and between juveniles and adults, indicating the presence of vocal learning [73]. Behavioral and/or neuroanatomical studies on most parrot species (with the exception of the budgerigar) including the endangered kea are few. Hence, additional experiments on volume measurements (beyond areas) with a larger number of animals from both sexes and from a wide variety of parrot species that differ in communication abilities would be necessary to further test these hypotheses on size differences.

In most species, the lowest core to shell area ratios (meaning shell is bigger) were in NLC. The NLC in budgerigars is required for production, but not memory of English words and natural vocalizations [74]. NLC lesions also disrupt the amplitude at which the carrier frequency of amplitude-modulated vocalizations are produced [74]; we note here that the lesions in that study included parts of both the NLC core and shell, making it difficult to pinpoint precise functional significance of the core and shell areas. Perhaps the strongest evidence regarding functional diversification of the NLC core and shell comes from a preliminary study by Striedter and Lei [75] where bilateral lesions of dorsal NLC (core in this study) and ventral NLC (shell in this study) were performed and learned contact calls examined. The authors found that NLC core lesions produced dramatic shifts in vocal frequency within a short period of time, with little recovery after lesions. However, NLC shell lesions produced a slow decrease in the size of the contact call repertoire. This suggests that further experiments are likely to discover different functions of the core and shell systems in parrot vocal communication.

Identification of core and shell functional differences is informed by the gene expression specializations. *SLIT1* belongs to a class of proteins involved in axon guidance and cell migration, and in a recent study we also showed that its mRNA is downregulated in the human laryngeal motor cortex [37], the analogue of AAC. The protein serves as a ligand to the *ROBO1* receptor [76], with a role in brain development [77], and is differentially upregulated by the human *FOXP2* transcription factor (involved in speech acquisition) more than the chimpanzee *FOXP2* [78]. Mutations in *ROBO1* cause speech sound disorders and dyslexia [79]. One potential hypothesis is that *SLIT1* is a candidate gene involved in the formation and maintenance of the specialized direct projection of the ACC core to brainstem vocal motor neurons [39]; *SLIT1* is mostly repulsive for forming connections, and its down-regulation could be a mechanism to allow a direct projection to form.

Although all nine parrot species examined showed *SLIT1* downregulation in the AAC core, a subset of them expressed intermediate specialized levels of *SLIT1* in the *PVALB*-defined shell that includes non-vocal motor areas. Together with the limited specializations of *mENK* and TH noted here in the adjacent motor regions of this or other parrot song nuclei suggest that the brain areas involved in movement control may also have some specialized connectivity and functions. Only vocal learning birds (parrots in particular) and mammals (humans, elephant, and a sea lion [said to be a non-learner exception among pinnepeds although not demonstrated]) show auditory-motor entrainment, synchronizing body movements to the rhythm of the downbeat in music (i.e. dance to music heard) [16,80–82]. Similar types of movements in budgerigars are associated with activation of the motor regions adjacent to the song nuclei shells [35] as noted here. Thus, it is tempting to speculate that the gene expression specializations in the non-vocal motor regions adjacent to the song nuclei could be related to their more unique ability to learn to dance. This hypothesis can be tested in experiments targeting manipulations of the specialized gene expression part of the adjacent motor brain regions.

The differential regulation of *FOXP1* in the NLC core and shell, and *NR2A* and *GLUR1* genes in the core, could mean that the downstream consequences on the genes that they target (*FOXP1’s* direct regulation or *NR2A’s* and *GLUR1’s* indirect single transduction regulation of genes) and their ultimately influence on behavior would be different than in the surrounding regions. The higher levels of gene products with functions in Ca2+ buffering, neuroprotection and cell metabolism in all the cores (*PVALB*, *NADPH-d*, and *CGRP*), suggest that the cores could be more active than the surrounds [38], as in songbird song nuclei compared to surrounding non-song areas.

Interestingly, except for higher *DUSP1* in MO and NAO cores, there is no noticeable difference in the relative degree of vocalizing-driven *EGR1 or C-FOS* mRNA induction between the core and shell region of any song nucleus in budgerigars. One explanation could be that both core and shell regions are equally required for production of warble song. Alternatively, IEG mapping techniques could be inadequate to parse out more finely tuned functions of the core and shell regions in vocal production. It is also possible that behavioral experiments that are designed for parrots to perform more specific vocal behaviors would be required to answer these questions. In either case, future IEG and other functional studies can now be guided by our discoveries to help determine potential functional differences between the core and shell regions.

In conclusion, we believe our study presents a new and viable hypothesis concerning the nature of unique specializations that characterize the parrot song system, which distinguish it from those of other vocal learning species. This hypothesis could inform future studies aimed at understanding the mechanisms of mimicry of human speech and the neural substrates that govern motor imitation in parrots, and provide a basis for deepening our understanding of the neural underpinnings of vocal learning.

## Methods

### Tissue collection and sectioning

We obtained brains from budgerigars (*Melopsittacus undulatus*) (n = 5; 3 males, 2 females) from the University of Copenhagen, Denmark colony, and from Duke University, USA (n = 25, both males and females that were also used in other studies); peach-fronted conures (*Aratinga aurea*) (n = 5; 3 males, 2 females), cockatiels (*Nymphicus hollandicus*) (n = 5; 3 males, 2 females), and peach-faced lovebirds (*Agapornis roseicollis*) (n = 5; 3 males, 2 females) from the University of Copenhagen; a male yellow lored Amazon parrot (*Amazona xantholora*), a male yellow crowned Amazon parrot (*Amazona ochrocephala*), a female blue and gold macaw (*Ara ararauna*) and a female kea (*Nestor notabilis*) from the Denmark Zoo (Copenhagen Zoo, Frederiksberg, Denmark); and a female African Grey parrot (*Psittacus erithacus*) from the Diagnostic Pathology Laboratory of the Dutch Research Institute for Avian and Exotic Animals (NOIVBD, Netherlands). The birds in the University of Copenhagen colony were briefly anaesthetized with CO_2_, whereas the birds collected from the Copenhagen Zoo were anaesthetized with sevoflurane and pentobarbital IV, or with medetomidine and ketamine before decapitation. The remaining birds used for behaviorally regulated gene expression were not anaesthetized prior to decapitation, in order to not disrupt the behaviorally-regulated gene expression. All collected brains were dissected within minutes of decapitation, frozen immediately in a dry ice-ethanol bath, and stored at -80°C. Brains were kept frozen on dry ice during transportation. We embedded the frozen brain in Tissue-Tek OCT Compound (Sakura, Finetek, Torrance, CA) in a plastic block mold and quickly froze the Tissue Tek around the brain on a dry ice-ethanol bath. Brains were sectioned in sagittal and coronal planes at 16μm in 6–20 series on a cryostat and the sections mounted on plus charged slides (Fisher Scientific, Pittsburgh, PA, USA). Only the coronal plane sections are shown, where it was easier to identify core and shell regions.

### Ethics statement

All procedures on live animals were approved by the Institutional Animal Care and Use Committee of Duke University and University of Copenhagen. Tissue collection and export were approved by the Tissue Collection permits of Denmark and Netherlands, and for import by the United States Department of Agriculture (Permit # 119548).

### Radioactive in situ hybridization

To localize mRNA expression of the *PVALB* and *SLIT1* genes we used radioactive ^35^S *in situ* hybridization following previously described procedures [54,83]. To make the *PVALB* riboprobe we used a clone from our zebra finch full-length cDNA collection [38, 46] (GenBank Accession # DQ215755), and a *SLIT1* clone containing 1.8Kb of the coding region that we cloned [39] (GenBank Accession # KF738084). Sections were fixed in 3% paraformaldehyde, rinsed in 1X phosphate-buffer saline (PBS), dehydrated in an increasing ethanol series, and then air-dried. We generated radioactive ^35^S-UTP-labeled sense and antisense riboprobes by reverse transcription, and hybridized each slide containing brain sections with 1x10^6^ cpm at 60°C (*PVALB*, *SLIT1*; the lower than 65°C temperature is to obtain cross-species hybridization). We exposed the slides to NTB emulsion (Kodak, USA) diluted 1:1 in distilled water for ~14–30 days at 4°C and processed the slides with D-19 developer (Kodak) and fixer (Kodak). We visualized the bound riboprobe as silver grains, and the cell bodies by counterstaining with cresyl-violet acetate solution (Sigma, USA). Tissue incubated with the sense probe showed no significant signal above background.

### Quantification of song nuclei size ratios

To perform quantification of area measurements, we captured digital images of brain sections of emulsion dipped (in darkfield) plus Nissl-stained (in brightfield) slides using an Olympus MVX10 microscope (Olympus, Japan) connected to a DP71 camera (Olympus) and DP Controller software. We quantified the core and shell area boundaries from all available sections from each animal per species. However, to remove any size measurement biases due to the number of available sections, we selectively analyzed the core and shell regions in sections where the core region was the largest, in 3–4 adjacent sections per brain region in each animal. We measured the area sizes using the Freehand tool in the Fiji software from ImageJ with the scale bar set in millimeters for spatial calibration. *PVALB* expression specializations and cresyl violet were used to visualize the posterior song nuclei (NLC and AAC) core and shell regions and the anterior song nuclei (MO and NAO) core regions, whereas only cresyl violet was used to visualize the shell regions of MO and NAO, as *PVALB* does not distinguish their shell boundaries. All statistical analyses were performed using IBM SPSS Statistics for Macintosh (Version 22.0).

### Analysis of connectivity of song nuclei in budgerigars

To investigate connectivity of the core and shell regions of NLC, AAC, MO, and NAO, we imaged and analyzed slides (> 4000) containing serial brain sections of budgerigars (n = 29) from neural tracing studies previously conducted in the Brauth lab [31,34] and compared them to serial brain sections of gene expression studies previously conducted in the Jarvis lab [24,35,38,44,45,54,84]. These include budgerigar brains with focal injections of 10–15% BDA in saline, and Biocytin into the different parts of what we have identified as NLC, AAC, MO, and NAO core and shell (Table 3). These serial sections included many previously unpublished images. We took digital images using a described procedure [46]. For NAO, we could not image brain sections with focal injections of fluorescent tracers [34] because the tissue still available had been stained with cresyl violet, which removed the tracer; thus, we examined the published study [34] to find examples of focal injections within NAO that we identified here as core and shell regions.

## Supporting Information

S1 Fig Brightfield photomicrographs (cresyl violet stained) of brain sections showing the AAC and NLC song nuclei in a budgerigar brain. (**a**) Boundaries of the NLC and AAC song nuclei consisting of core and shell regions (delineated by dashed black lines); (**b**) Boundaries of the core region of NLC; (**c**) Boundaries of the core region of AAC; (**d–f**) Views (from low power to high power) of tightly packed clusters of cells arranged in a sphere like shape comprising the core region of NLC, in a black and white setting under brightfield; (**g–i**) Views of widely dispersed clusters of cells in the shell region; (**j–l**) Views of closely packed cells in the core region of AAC; (**m–o**) Views of widely dispersed cells in the shell region of AAC. The shell boundaries in these images are based on the *PVALB* expression, and thus include the non-vocal motor areas. Sections are in the coronal plane; medial is to the left, dorsal is top.
(TIF)

S2 Fig Brightfield photomicrographs (cresyl violet stained) of brain sections showing the MO and NAO song nuclei in a budgerigar brain. (**a**) Boundaries of the MO song nucleus consisting of core and shell regions (delineated by dashed black lines); (**b**) Boundaries of the NAO song nucleus consisting of core and shell regions (delineated by dashed black lines); (**c**) Schematic diagram showing relative positions of MO and NAO song nuclei; (**d–f**) Views (from low power to high power) of large loosely distributed cells arranged like a sphere in the MO core region; (**g–i**) Views of the shell region of MO consisting of widely distributed cells; (**j–l**) Views of closely packed cells of the NAO core region; (**m–o**) Views of widely distributed cells in the shell region of NAO. Sections are in the coronal plane; medial is to the left, dorsal is top.
(TIF)

## Anatomical Abbreviations

A: arcopallium
AAC: central nucleus of the anterior arcopallium
AACd: dorsal part of the central nucleus of the anterior arcopallium
AACv: ventral part of the central nucleus of the anterior arcopallium
ACM: caudal medial arcopallium
AH: anterior hyperpallium
Ai: intermediate arcopallium
AMV: anterior ventral mesopallium
AN: anterior nidopallium
Area X: a vocal nucleus
AR: androgen receptor
Av: nucleus avalanche
B: basorostralis
Cb: cerebellum
CM: caudal mesopallium
CSt: caudal striatum
CMM: caudomedial mesopallium
DLN: dorsal lateral nidopallium
DLM: dorsal lateral nucleus of the thalamus
DM: dorsal medial nucleus of the midbrain
DMm: magnocellular nucleus of the dorsal thalamus
E: entopallium
GP: globus pallidus
H: hyperpallium
Hp: hippocampus
HVC: a vocal nucleus (no abbreviation)
HVo: oval nucleus of the ventral hyperstriatum
HVoc: HVo complex
IH: intercalated hyperpallium
IEG: immediate early gene
LAI: lateral intermediate arcopallium
LAN: lateral nucleus of the anterior nidopallium
LAM: lateral nucleus of the anterior mesopallium
LMAN: lateral part of the magnocellular nucleus of the anterior nidopallium
M: mesopallium
MAN: magnocellular nucleus of the anterior nidopallium
MLd: dorsal part of the lateral mesencephalic nucleus
MMSt: magnocellular nucleus of the medial striatum
MO: oval nucleus of the anterior mesopallium
MD: dorsal mesopallium
MV: ventral mesopallium
nXIIts: 12^th^ nucleus, tracheosyringeal part
N: nidopallium
NAo: oval nucleus of the anterior neostriatum
NAO: oval nucleus of the anterior nidopallium
NAoc: NAo complex
NAom: medial division of the oval nucleus of the anterior neostriatum
NAs: supralaminar area of the frontal neostriatum
NCM: caudomedial nidopallium
NDC: caudal dorsal nidopallium
NIDL: dorsal lateral intermediate nidopallium
Nif: interfacial nucleus of the nidopallium
NLc: central nucleus of the anterior neostriatum
NLC: central nucleus of the lateral nidopallium
NLs: supracentral nucleus of the lateral nidopallium
NLv: ventral lateral nidopallium
TeO: optic tectum
Ov: nucleus ovoidalis
PH: posterior hyperpallium
RA: robust nucleus of the arcopallium
Rt: nucleus rotundus
SLN: supra lateral nidopallium
St: striatum
VA: vocal nucleus of the arcopallium
VAM: vocal nucleus of the anterior mesopallium
VAN: vocal nucleus of the anterior nidopallium
VANp: posterior part of the vocal nucleus of the anterior nidopallium
VASt: vocal nucleus of the anterior striatum
VMM: vocal nucleus of the medial mesopallium
VMN: vocal nucleus of the medial nidopallium
VLN: vocal nucleus of the lateral nidopallium
