# Supplementary figures and images for: Core and Shell Song Systems Unique to the Parrot Brain
Source: PLoS One. 2015 Jun 24;10(6):e0118496. doi: 10.1371/journal.pone.0118496 (PMC4479475; doi:10.1371/journal.pone.0118496)

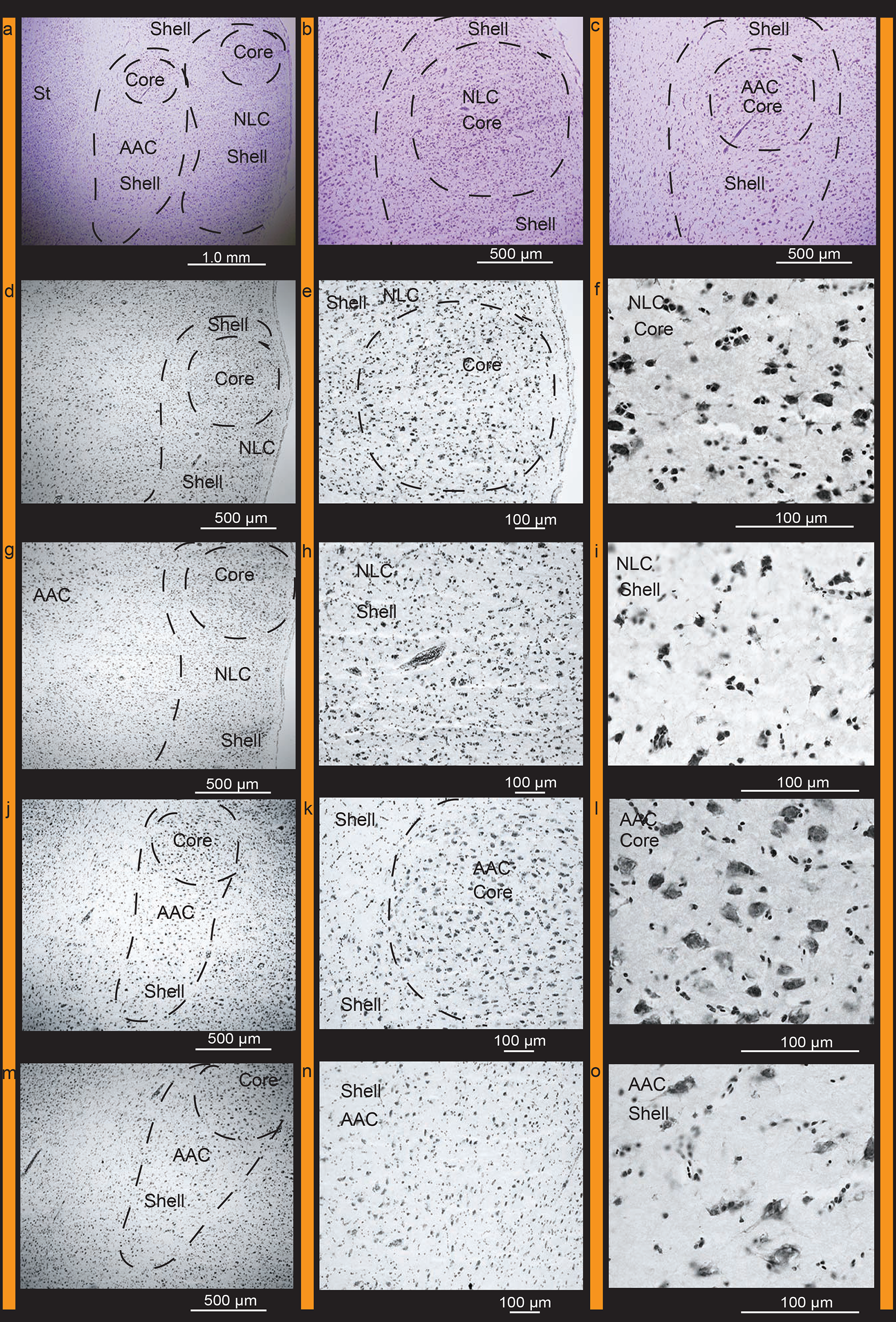

Supplement: S1 Fig — (a) Boundaries of the NLC and AAC song nuclei consisting of core and shell regions (delineated by dashed black lines); (b) Boundaries of the core region of NLC; (c) Boundaries of the core region of AAC; (d–f) Views (from low power to high power) of tightly packed clusters of cells arranged in a sphere like shape comprising the core region of NLC, in a black and white setting under brightfield; (g–i) Views of widely dispersed clusters of cells in the shell region; (j–l) Views of closely packed cells in the core region of AAC; (m–o) Views of widely dispersed cells in the shell region of AAC. The shell boundaries in these images are based on the PVALB expression, and thus include the non-vocal motor areas. Sections are in the coronal plane; medial is to the left, dorsal is top. (TIF) [file pone.0118496.s001.tif]

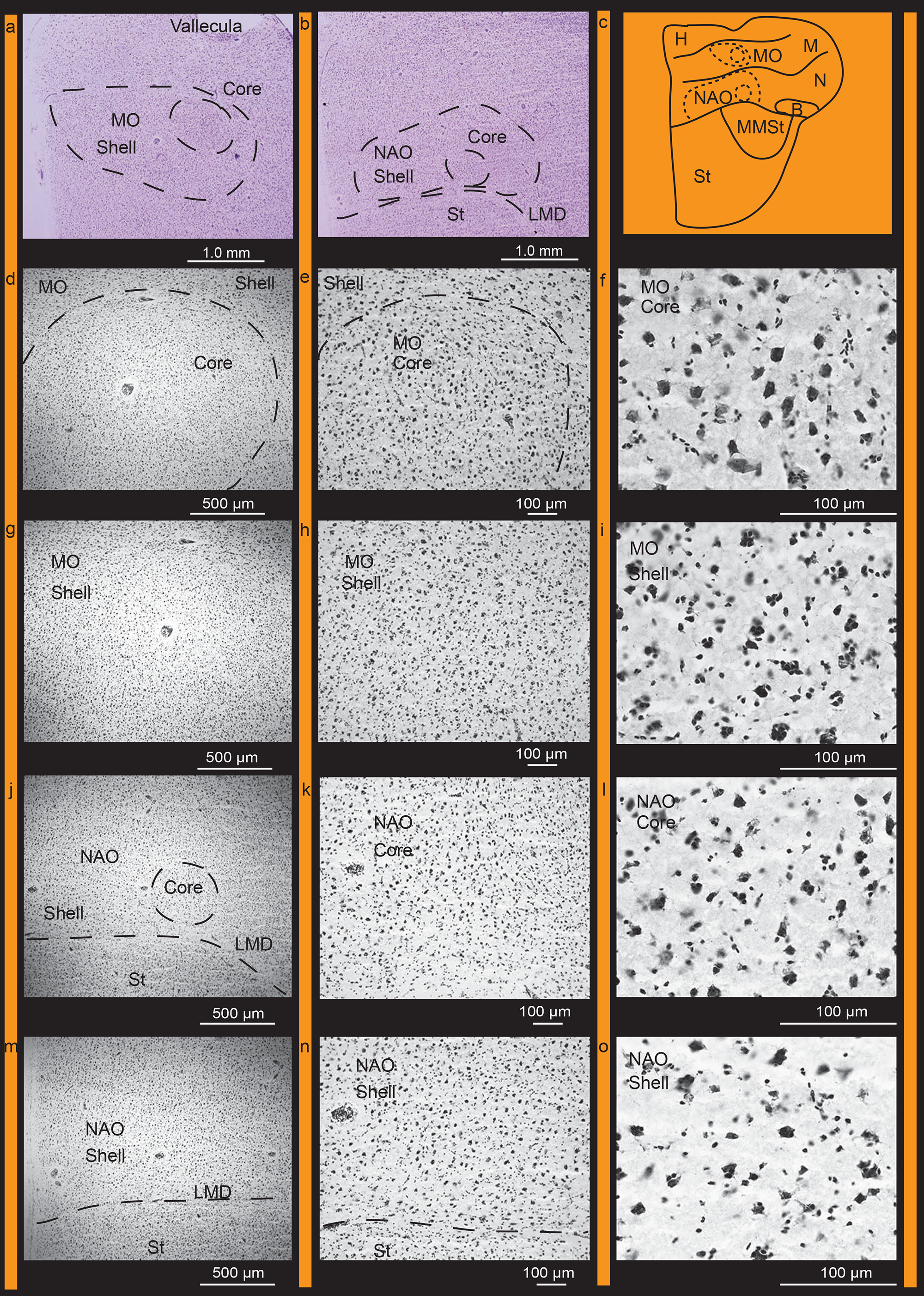

Supplement: S2 Fig — (a) Boundaries of the MO song nucleus consisting of core and shell regions (delineated by dashed black lines); (b) Boundaries of the NAO song nucleus consisting of core and shell regions (delineated by dashed black lines); (c) Schematic diagram showing relative positions of MO and NAO song nuclei; (d–f) Views (from low power to high power) of large loosely distributed cells arranged like a sphere in the MO core region; (g–i) Views of the shell region of MO consisting of widely distributed cells; (j–l) Views of closely packed cells of the NAO core region; (m–o) Views of widely distributed cells in the shell region of NAO. Sections are in the coronal plane; medial is to the left, dorsal is top. (TIF) [file pone.0118496.s002.tif]
